# Facilitators and Barriers to Lung Cancer Screening during Long COVID: A Global Systematic Review and Meta-Study Synthesis of Qualitative Research

**DOI:** 10.3390/ijerph21050534

**Published:** 2024-04-25

**Authors:** Teferi Gebru Gebremeskel, Frank Romeo, Adisu Tafari Shama, Billie Bonevski, Joshua Trigg

**Affiliations:** 1 Flinders Health and Medical Research Institute (FHMRI), College of Medicine and Public Health, Flinders University, Bedford Park, P.O. Box 2100, Adelaide, SA 5001, Australia; billie.bonevski@flinders.edu.au (B.B.);; 2 Department of Reproductive Health, College of Health Sciences, Aksum University, Aksum P.O. Box 1010, Tigray, Ethiopia; 3 S.H.R.O SBARRO Organization, College of Science and Technology, Temple University, RM 00196 Roma, Italy; 4 Department of Public Health, Health Institute, Wollega University, Nekemte P.O. Box 395, Wollega, Ethiopia; adisuteferi1906@gmail.com

**Keywords:** facilitators, barriers, lung cancer, screening, long COVID, worldwide

## Abstract

**Background**: Participation in targeted screening reduces lung cancer mortality by 30–60%, but screening is not universally available. Therefore, the study aimed to synthesize the evidence and identify facilitators and barriers to lung cancer screening participation globally. **Methods**: Two reviewers screened primary studies using qualitative methods published up to February 2023. We used two-phase synthesis consistent with a meta-study methodology to create an interpretation of lung cancer screening decisions grounded in primary studies, carried out a thematic analysis of group themes as specific facilitators and barriers, systematically compared investigations for similarities and differences, and performed meta-synthesis to generate an expanded theory of lung cancer screening participation. We used the Social Ecological Model to organize and interpret the themes: individual, interpersonal, social/cultural, and organizational/structural levels. **Results**: Fifty-two articles met the final inclusion criteria. Themes identified as facilitating lung cancer screening included prioritizing patient education, quality of communication, and quality of provider-initiated encounter/coordination of care (individual patient and provider level), quality of the patient–provider relationship (interpersonal group), perception of a life’s value and purpose (cultural status), quality of tools designed, and care coordination (and organizational level). Themes coded as barriers included low awareness, fear of cancer diagnosis, low perceived benefit, high perceived risk of low-dose computerized tomography, concern about cancer itself, practical obstacle, futility, stigma, lack of family support, COVID-19 fear, disruptions in cancer care due to COVID-19, inadequate knowledge of care providers, shared decision, and inadequate time (individual level), patient misunderstanding, poor rapport, provider recommendation, lack of established relationship, and confusing decision aid tools (interpersonal group), distrust in the service, fatalistic beliefs, and perception of aging (cultural level), and lack of institutional policy, lack of care coordinators, inadequate infrastructure, absence of insurance coverage, and costs (and organizational status). **Conclusions**: This study identified critical barriers, facilitators, and implications to lung cancer screening participation. Therefore, we employed strategies for a new digital medicine (artificial intelligence) screening method to balance the cost–benefit, “workdays” lost in case of disease, and family hardship, which is essential to improve lung cancer screening uptake.

## 1. Introduction

Lung cancer is the world’s number one cause of cancer death, exceeding deaths from breast, colon, and prostate cancers [1,2]. Lung cancer has become a significant concern in global public health, with its incidence showing a consistent upward trend [3]. This disease is highly prevalent and known for its aggressive nature. In 2020 alone, there were approximately 2.2 million new cases of lung cancer, and sadly, it resulted in 1.8 million deaths [4]. The mortality rate linked to lung cancer is particularly problematic due to its aggressive characteristics and the challenges in early detection, which further underscore the urgency of addressing this issue.

More than 50 percent of lung cancer diagnoses are made at an advanced stage, which makes this cancer challenging to treat, and the chance of survival is much lower [5]. Early diagnosis and participation in cancer screening programs are some of the most crucial factors in reducing cancer-related illness and mortality [6]. Understanding the obstacles and facilitators of lung cancer screening practices can help improve access to information about cancer screening, address lung cancer screening behaviors, improve communication between individuals and health professionals, and strengthen shared decision-making [7].

Primary prevention offers the most cost-effective long-term strategy for lung cancer control, even when combined with comprehensive efforts to reduce the burden of lung cancer, including secondary prevention or early detection of the disease at its early stage, which is crucial, such as proactive screening programs [8,9]. Lung cancer screening is an essential tool in early cancer diagnosis for high-risk groups, such as those who smoke tobacco, to decrease their chance of dying from the persistent risk of lung cancer [10].

While the COVID-19 pandemic has shed light on health disparities in our most vulnerable communities, it has reduced the identification rate of lung cancer cases, as worries and fears have prevented important healthcare-seeking behaviors, such as visiting healthcare professionals [5]. Some COVID-19 public health measures, such as social distancing and quarantine, may also have impacted cancer screening behaviors [11]. Now more than ever, it is essential for healthcare professionals to talk to individuals about lung cancer screening and particularly to people at risk of lung cancer and advise them to contact their healthcare professionals to arrange a screening session for lung cancer [5].

Low-dose computerized tomography (LDCT) is recommended to reduce mortality for people at high risk of developing lung cancer [12]. Evidence shows that nearly 8 million Americans are at increased risk for lung cancer and could benefit from LDCT-based lung cancer screening, but only 5.7 percent are screened [13].

Several barriers significantly inhibit participation in lung cancer screening. These include poor patient and provider awareness, challenges in identifying, enrolling, and tracking lung cancer screening patients, and disparities in insurance coverage, stigma, and quality incentives at the health system level [14,15,16,17,18,19].

Globally, lung cancer screening is not consistently offered nationally, with current provisions taking the form of local pilots and programs in Europe and a lung cancer screening inquiry in Australia to examine national expectations, processes, and conditions [20,21]. Additionally, in the USA, a lung cancer diagnosis is often based on the physician’s support [21].

Previous research has mainly focused only on sex-based and social differences of participants in lung cancer screening programs. In addition, the abovementioned research used similar risk factors to identify the barriers and facilitators. However, previous research has yet to include the impacts of COVID-19 as a factor. There is still a lack of evidence to systematically map the barriers to screening that have implications for lung cancer screening uptake and identify facilitators for effective lung cancer screening. Our review is the first systematic review of qualitative studies to summarize critical barriers and facilitators relevant to the general screening population, primary care practitioners, social or cultural, and lung cancer screening programs. Therefore, the study aimed to synthesize the evidence and identify facilitators and barriers to lung cancer screening participation globally.

## 2. Methods

### 2.1. Study Design

A systematic review and qualitative synthesis were conducted to assess the barriers and facilitators of lung cancer screening and were registered in PROSPERO (CRD42022363115) [8].

### 2.2. Search Strategy

In the literature search, articles were considered eligible if they were published between September 2000 and February 2023. Six databases, Psych INFO, CINAHL, Web of Science, PubMed, SCOPUS, and the Cochrane Library, were used (1980–2023) were searched to identify studies addressing the barriers and facilitators of lung cancer screening. Google Scholar searches and manual extraction of relevant articles were also used to search for additional literature on the publication reference lists. The search terms included “((lung cancer) AND (screening)) AND (facilitator or barrier)”.

The search identified 3356 studies using the entire search string, as listed in Supplementary Table S1. In addition, we followed the Preferred Reporting Items for Systematic Reviews and Meta-Analyses (PRISMA) guidelines when reporting the literature search [22]. We used the Enhancing Transparency in Reporting the Synthesis of Qualitative Research (ENTREQ) checklist to guide reporting of review outcomes [23] (Tables S2–S5).

### 2.3. Inclusion and Exclusion Criteria

All retrieved studies were imported into an endnote library to exclude duplicates or irrelevant titles unrelated to lung cancer screening. Next, we performed identification and abstract screening to confirm that the articles met the methodological criteria and ensured that the reports were published in English (Table 1). This resulted in 95 articles that met the eligibility criteria. Finally, we included the full text of all articles that met the inclusion criteria for a final total of 52 articles.

### 2.4. Data Extraction and Synthesis

All de-duplicated titles and abstracts were screened by two authors (TGG and ATS) based on the eligibility criteria. They included studies using the guidelines and the Critical Appraisal Skills Program (CASP) tools [23]. We also assessed the study’s validity (study quality assessment) and verification of the extracted information. The preferred reporting Items for Systematic Reviews and Meta-Analyses (PRISMA) Structured Guidelines and Checklist were followed to present this article as a narrative review. We used a custom Microsoft Excel template to extract qualitative data from all relevant articles. The template contains the first author’s name, year of publication, country, study purpose, setting, socio-demographics, sample, design, analysis methods, primary findings, and study conclusions and implications.

### 2.5. Data Analysis

Following the meta-study methodology, we employed a two-stage data synthesis method to derive an interpretation of lung cancer screening decisions from the original studies [24,25]. In the first phase, we focused on the interpretive analysis of research findings by categorizing group themes as specific facilitators and barriers and systematically comparing studies for similarities and differences between themes [26]. Secondly, we examined how the methodological gaps in the quality of the included studies affected the interpretation of findings. For example, information in terms of the weaknesses and strengths of the study increases our understanding of the reliability and integrity of the evidence, strengthening our interpretations. Finally, we examined how theory in primary studies influenced our understanding of findings.

In the second phase, in-depth data synthesis, based on the primary studies in this review and the triangulation of findings from the three steps in Phase 1, was performed, and we provided an interpretive framework that captures key facilitators and barriers that influence participation in lung cancer screening decisions and strategies for patients and providers. We interpreted the authors’ reporting of the data, the study quality, and the theoretical frameworks or perspectives used in the research.

To help organize and interpret the data, we used the Social Ecological Model [24], a well-studied behavioral health theoretical model that explains how an individual’s relationships and environment can influence health behaviors. The Social Ecological Model (SEM) includes four primary levels: (i) the social/cultural level (related to social/cultural norms and a patient’s health determinants), (ii) the organizational level (institutions that have the structural capacity to promote health, (iii), the interpersonal level (related to the patient’s relationship to provider), and (iv) the individual level (related to the patient’s knowledge) [24]. The relationships between each level and the influencers within the same level are equally important [24].

## 3. Results

**Figure 1:**
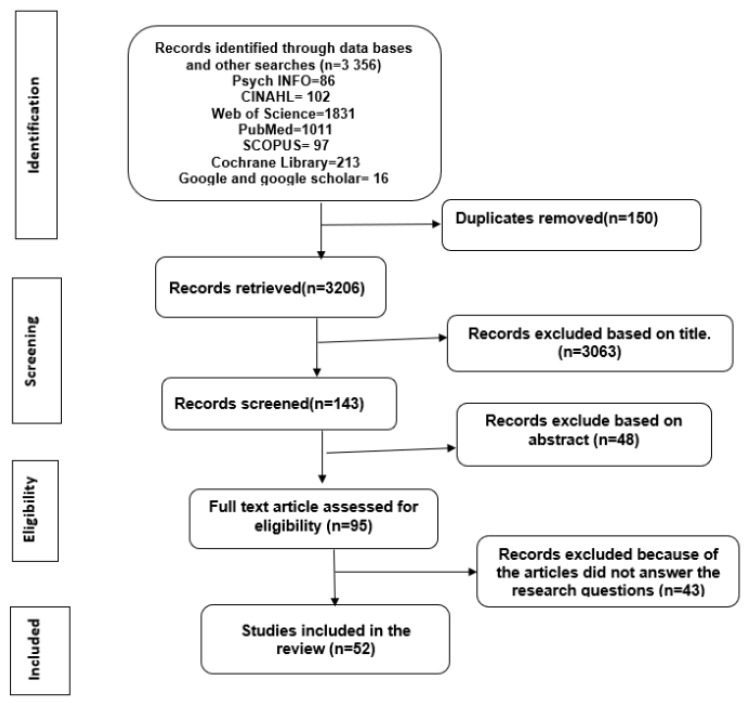
PRISMA flow diagram for screening process and outcomes.

Fifty-two articles were included in the final review of the ninety-five articles found via full-text abstract screening (Figure 1). Studies reported experience from participants in nine countries, most in the USA (n = 32), with the remainder in the UK (n = 9), Australia (n = 3), the Netherlands (n = 2), Canada (n = 2), and one each in India, Turkey, New Zealand, and South Asia.

### 3.1. Meta-Data Analysis

Our findings revealed several themes across facilitator and barrier factors and stratified into four sections (individual, interpersonal, cultural, and organizational). Themes facilitating lung cancer screening participation were individual patient and provider level (prioritizing patient education, quality of communication, and quality of provider-initiated encounter/coordination of care), interpersonal status (quality of the patient-provider relationship), and its social or cultural level (perception of a life’s value and purpose), organizational level (quality of tools designed and care coordination), and the converse of these were barriers to lung cancer screening Table S1.

### 3.2. Quality Appraisal (Meta-Method Analysis)

We examined studies guided by the Critical Appraisal Skills Program (CASP) [23] tool to assess the quality of qualitative analyses. Based on the CASP criteria, the average quality score for the 52 included studies was 8 out of 10 (range 3–10). Of those included studies, 45 (86 percent) of them met at least 8 out of 10 of the Critical Appraisal Skills Program criteria, and 6 (11 percent) met from 4 to 7 standards (Tables S2–S5).

Our meta-method showed that 70% of all review articles identified at least 8 of 10 Critical Assessment Skills Program criteria as barriers or facilitators to lung cancer screening participation, with 50% identifying three or fewer of the Critical Assessment Skills Program criteria.

According to the meta-method analysis, most of the reviewed articles demonstrated coherence between the research question, methods, analysis strategy, and presentation of results. Of the total studies reviewed, 40 (77 percent) of the studies addressed the sampling strategy and details of the study participants appropriately to increase the transferability of findings to similar populations, and 44 (85 percent) of the studies used appropriate qualitative research designs for their research.

### 3.3. Framework Model or Theory (Meta-Theory Analysis)

Of the total review articles, 17 articles (33%) used a framework or theoretical model, primarily the health belief model [27], to guide their investigation, and we assessed how this influenced the choice of data collection and interpretation [28,29,30,31,32,33,34,35,36,37,38,39,40,41]. The remaining 35 studies (65%) did not use a framework or theoretical model to guide their investigation. They assessed how this influenced the choice of data collection and interpretation. Still, it was suggested that most of the identified determinants informed their research questions and guidelines for lung cancer screening from their literature.

### 3.4. Facilitators of Lung Cancer Screening Participation

Individual and interpersonal facilitators. Studies showed that individuals who were aware of early detection lung cancer screening [21,22,23,24,25,26,27,28,29,30,31], high perceived benefit [30,42,43,44], motivation to quit smoking [32,33,38,45], provision of the mobile testing program and home test kits during COVID-19 [46], enthusiasm for lung cancer [28,32,33,34,38,42,43,45,47,48,49,50], a screening recommendation from a healthcare provider [30,33,38,45,51], and shared decision-making interaction between discussion [31,52,53,54] were more supportive of screening and tended to be screened Table S1.

For example, in a qualitative study conducted by Lowenstein et al. involving 42 screening-eligible patients, “it was discovered that participants expressed positive attitudes towards screening. They found the concept of screening acceptable and associated it with the notions of prevention and early detection” [48].

Social and organizational facilitators. Studies indicated that reduced costs in specific facilities (some country-qualified, already established healthcare settings) [30,35,55], the value of life and perceptions of age, and altruism [54,56] were more supportive of screening and tended to be screened Table S1.

In a qualitative study by Klarenbeek, S.E. [35], it was suggested that involving the managing directors of all relevant medical departments is crucial. The study highlighted the potential financial burden on these departments due to the system being implemented. Transparency regarding these costs is essential to gaining support from management.

### 3.5. Barriers to Lung Cancer Screening Participation

Individual and interpersonal barriers. Studies showed that low awareness of lung screening [28,30,31,32,33,36,37,38,39,41,44,45,48,50,57,58,59], fear of cancer diagnosis, and worry [31,32,33,36,37,38,40,43,45,46,47,48,54,58,60,61], low perceived benefit (feeling healthy) [30,37,48,62], concern or high perceived risk of LDCT [28,33,34,38,41,42,44,45,47,49,60], COVID-19 fear (perceptions of risk, mortality, worry, behavioral and psychosocial responses to COVID-19) [61,63,64,65,66], anxiety of causing misunderstanding during a risk–benefit conversation [48,52], patient education (provider recommendation) [30,37,42,47,49,51,57,62,67], patient misunderstanding [36,50,62], and inadequate time [29,30,52,55,68] served as barriers to seeking screening Table S1. For example, a study of knowledge from high-risk communities and providers has shown that “a significant number of high-risk individuals have never heard of screening or that a primary care practitioner has never introduced the concept of screening to them” [31,38].

Social and organizational barriers. Findings indicated that distrust in the service/health professional [28,31,32,33,34,37,60,67], fatalistic beliefs [28,31,33,36,42,45,48,60], perception of aging [36,37], costs and copays [28,30,37,43,51,55,67], lack of insurance coverage [38,51,53], inadequate infrastructure [35,44,51,55,69], lack of care coordinators [44,56,67], and lack of institutional policy [35,44,54,69] served as barriers to seeking screening. For example, “Hoffman et al. studied PCPs in New Mexico and found that limited knowledge of guidelines and skepticism towards high false-positive rates posed significant barriers for providers” [70]. Table S1 shows the identified barriers.

### 3.6. Meta-Synthesis of Themes

Our review found several barriers and facilitators influencing the decision to undergo lung cancer screening in eligible patients. Based on the primary studies in this review and the triangulation of findings from the three steps in Phase 1, we provide an interpretive framework that captures key facilitators and barriers that influence participation in lung cancer screening decisions and strategies for patients and providers.

This review, combined with overcoming other structural and motivational barriers to screening, found that individual awareness may be essential to screening people for lung cancer (Figure S1). This review found that high knowledge of lung cancer screening was a crucial factor in engaging people in the context of cancer screening, while those with low knowledge were less likely to engage in screening. Individuals’ lack of awareness of lung cancer screening was associated with negative perceptions of cancer and unrealistic fear and fatalism but independently acted as a barrier to screening uptake. In contrast, appropriate awareness resulted in positive views, creating motivation for individuals to participate in screening.

The interpersonal (provider–patient) level (e.g., provider recommendation, inadequate encounter time), social/cultural level (e.g., distrust in the service/health professional, fatalistic beliefs), and organizational/institutional policies (e.g., costs and copays, lack of insurance coverage, lack of care coordinators, lack of institutional approach) were the most significant factors influencing the decision to participate in lung cancer screening among specific population groups with lower participation.

## 4. Discussion

We explored the facilitators and barriers towards lung cancer screening in the context of individual (patient), interpersonal (provider–patient), social (cultural), and organizational (institutional policy) levels. Our review is the first systematic review of qualitative studies to summarize critical barriers and facilitators relevant to the general screening population, primary care practitioners, social and cultural factors, and lung cancer screening programs. Our analysis showed that although some barriers and facilitators to lung cancer screening are shared across contexts, most studies included diverse populations, designs, and methods. 

Our review synthesis showed that an essential factor influencing the decision to participate in lung cancer screening is “awareness”, including knowing where to access services (i.e., service location), assumptions about the screening process, the need to screen in the asymptomatic state, and having inaccurate information about what a lung cancer screening result means.

The literature showed that appropriate awareness was a requisite for participation in lung cancer screening. It was integral in overcoming other reported cultural, social, and structural or organizational policy barriers to screening. In our review, participants’ lack of awareness of lung cancer screening was frequently reported, i.e., in 17 articles [28,30,31,32,33,36,37,38,39,41,44,45,48,50,57,58,59]. Participants often had not heard of lung cancer or chose not to be screened due to fear of a cancer diagnosis or concerns about cost, which affected their attitudes about lung cancer and their motivation to participate in lung cancer screening. For example, a study of knowledge from high-risk communities and providers showed that a significant number of high-risk individuals have never heard of screening or that a primary care practitioner has never introduced the concept of screening to them [38].

Our review acknowledged that primary care practitioners need more screening knowledge in test sites, eligibility, criteria, methods, and insurance coverage. Four semi-structured interview studies discovered many primary care practitioners with current guidelines and a high false positive rate. There were challenges with limited knowledge among primary care providers [29,52,53,55]. Comparisons can be drawn with breast screening, where women were concerned about the accuracy of the test [71].

Although it is essential to address concerns about cancer itself or the high perceived risk of low-dose CT scans and to provide adequate information and opportunities to discuss these critical issues, the practical barriers to screening for lung cancer far outweigh the benefits in the literature reviewed [31,32,33,34,38,42,47,49,50,60]. However, these concerns and other factors may be enough to encourage someone to participate.

People at higher risk of developing lung cancer, fear of infection, the perceived risk of exposure to COVID-19, mortality, worry, and behavioral and psychosocial responses to COVID-19 were pervasive factors in the qualitative literature on screening [61,63,64,65,66]. Lung cancer counseling and community education pre-screening and post-screening through the media and other outlets that address these fears can help make communities aware of their role with individuals undergoing lung screening.

Fear of cancer diagnosis was a prevalent factor in the qualitative literature surrounding screening. Along with the fear of invasive procedures—psychological trauma associated with cancer anxiety and waiting for screening results—they are suggested to prove significant barriers to screening participation and interpersonal relationships. This finding reflects decisions to help-seeking for cancer signs and symptoms, which may help predict behaviors regarding lung screening involvement [72,73].

The shared decision-making process and decision-making aids were considered significant facilitators of participation in lung cancer screening and influenced their views of it among included studies [31,52,53,54]. However, Wiener’s investigation of the Adherence to annual lung cancer screening in a diverse population showed that most providers inconsistently incorporated decision aids [74].

This review found that the quality of patient–provider communication plays a crucial role in addressing barriers and facilitators to lung cancer screening participation. Our study provides additional insights into a provider’s ability to deliver information that may have the most influence. For example, if it is complicated to explain possible false positives, manage a patient’s worries, and when patients with low health literacy reported having difficulties understanding medical terms used during health professional recommendations [30,37,42,47,49,51,57,62,67]. In many cases, PCP did not make the conversion about lung cancer screening to high-risk people, and when they did, out of fear of causing a misunderstanding during a ‘risk–benefit conversation’ [36,50,62] or ‘ethical considerations’, the instructions provided were often insufficient [54,67] and the encounter time was inadequate [29,30,52,55,68]. Healthcare providers need to know how to work cancer screening into policies and procedures. Otherwise, it is not easy, and they will need more time for shared decision-making processes [70,75]. As shown in this review and other studies, the quality of patient–provider communication can significantly affect patients’ awareness, perception, and, ultimately, participation in lung cancer screening.

The influence of social or cultural beliefs on individuals’ decisions to participate in lung cancer screening has been demonstrated in many studies, and some patients’ belief systems prevent them from taking proactive participation in screening lung. Sharaf et al. [76] reported challenges that may adversely affect patients’ adherence to follow-up visits, referrals, and to primary care practice recommendations for lung cancer screening. Our review found specific barriers were prominent in social or cultural beliefs, such as distrust in the service/health professional [28,31,32,33,34,37,60,67], fatalistic beliefs [28,31,33,36,42,45,48,60], and perception of aging [36,37]. However, related to these beliefs is the issue of trust in the medical system. Our society operates at different levels of trust. Still, many patients need more trust in the medical system, including suspicion of health information, motives of doctors or other health authorities, primary care providers, and the entire screening process [28,43,76]. For example, Powell et al. examined the relationship between medical mistrust and delays in preventive health screenings and found that African-American men report higher medical distrust [77]. Further studies are needed to evaluate practical approaches to addressing medical mistrust and reducing delays in preventive health screenings due to perceived racism in healthcare.

One of the combined aims is to understand better organizational or structural policy barriers contributing to lung cancer screening participation inequities. Robust infrastructure, comprehensive cancer control centers, appropriate referral policies, and establishing a primary care provider network to monitor and control positive findings are needed at the system level to increase equitable lung cancer screening participation. For example, studies using focus group discussions discovered the number of risks of low-dose CT scans could impact a health system’s ability to increase equitable and effective lung cancer screening participation [78].

Our review revealed participants noted the inability to afford costs and the patient’s lack of insurance coverage for lung cancer screening. These findings are supported by a recent study of implemented programs examining the challenges of lung cancer screening among diverse and low-income outpatients, who reported a lack of understanding of the purpose of lung cancer screening and a need for personalized information focused on its benefits [70].

There is a need to improve structural or organizational systems and policies to ensure equitable access to lung cancer screening and the use of competent primary care providers and screening coordinators in the education process. The Centers for Medicare and Medicaid Services (CMS) implemented a merit-based incentive payment system (MIPS) program to compensate health systems for a variety of quality measures, such as breast, cervical, and colorectal cancer screening rates [65]. However, the merit-based incentive payment system (MIPS) program has not yet recognized lung cancer Screening as a preventative care measure. This prevents health systems from prioritizing lung cancer screening as much as breast, cervical, and colorectal cancer screening programs.

### 4.1. Limitation of This Review

Qualitative research may be contextually bound, transferable, and not generalizable. However, our findings show that combining qualitative assessments can provide more information and identify consistent results between studies and populations. Several studies support this. Personal views of lung cancer and screening participation may influence our understanding and interpretation of data. We performed an effective literature search to obtain the best available evidence, but there is still a risk that we may have missed some studies [79]. However, we utilized a research librarian who designed and implemented our key search strategy to address this concern.

### 4.2. Implications and Future Directions

The findings on individual-level and social/cultural barriers to lung cancer screening align with existing theories on health behaviors, especially the socioeconomic model. Individual-level barriers, such as lack of awareness, knowledge gaps, and concerns about screening, need comprehensive solutions to promote participation. Providing accurate and balanced information about screening risks and benefits is crucial. Social and cultural barriers, including distrust, fatalistic beliefs, and fear, also influence health behaviors and require culturally appropriate intervention programs. These findings support the need for comprehensive approaches considering individual, social, and cultural factors to improve the effectiveness and equity of lung cancer screening.

It is crucial to address organizational and institutional barriers to improve lung cancer screening and ensure equitable participation. These barriers include communication issues, decision-making challenges, transportation constraints, time limitations, costs and copays, infrastructure limitations, and institutional policies. These barriers should be considered and mitigated when designing a lung screening service. One strategy is implementing new digital medicine screening methods, such as artificial intelligence devices, for large prevention programs [80]. These systems can help identify individuals at higher risk of developing lung cancer, leading to cost savings by detecting and treating the disease earlier.

Additionally, digital screening methods can save money in reduced workdays, provide comfort to families, enable the creation of genetic databases for personalized therapy in the future, enhance quality, reduce costs, and assist physicians in decision-making. Policymakers should also establish clear screening guidelines and policies, encourage healthcare systems to adopt them, expand insurance coverage, invest in public education and awareness campaigns, collaborate with community organizations, support healthcare provider training and integration, foster collaboration, and care coordination, promote decision support tools, and implement mechanisms for monitoring and evaluating screening programs. By implementing these recommendations, policymakers can overcome barriers, improve access, and ensure informed decision-making in lung cancer screening. There is a lack of high-quality empirical evidence to support a specific strategy for lung cancer screening during COVID-19. However, adopting a theoretical screening model to understand patient-level barriers is recommended to maximize its benefits now and in the future.

## 5. Conclusions

Facilitators of lung cancer screening include individual factors such as awareness, recognizing benefits, motivation to quit smoking, access to mobile testing and home kits, enthusiasm, provider recommendations, and shared decision-making. Social and organizational facilitators include reduced costs, valuing life and age, and altruistic motivations. These factors promote a positive attitude towards screening and increase screening rates.

Individual barriers to lung cancer screening include low awareness, fear, worry, low perceived benefit, concerns about high risk, fear of COVID-19, and lack of patient education. Social and organizational barriers involve distrust in the healthcare system, fatalistic beliefs, perception of aging, financial obstacles, inadequate infrastructure, lack of care coordinators, absence of institutional policies, limited knowledge of guidelines, and skepticism among healthcare providers. Screening programs can target facilitators and barriers identified in this review to increase participation in lung cancer screening. For instance, educational interventions addressing awareness and overcoming logistical, interpersonal, and cultural barriers are effective strategies. Furthermore, programmatic approaches for a new digital medicine (artificial intelligence) lung cancer screening method are practical.

## Figures and Tables

**Table 1:** Eligible criteria of articles to ward lung cancer screening barriers and facilitators.

Characteristics	Inclusion Criteria	Exclusion Criteria
Publication time	All published Articles from January 1980 to February 2023 were included.	
Language	Articles published in English were included.	Articles published in languages other than English were excluded.
Type of Articles	All articles on lung cancer screening towards barriers and facilitators were included irrespective of the type of article and methodology.	Articles that did not identify barriers and facilitators toward lung cancer screening were excluded.

## Data Availability

All dataset(s) supporting the conclusions of this article are available upon reasonable request.

## Supplementary Materials

The following supporting information can be downloaded at: https://www.mdpi.com/article/10.3390/ijerph21050534/s1, Figure S1: A conceptual framework of factor influence to participate lung cancer screening; Table S1: Themes, according to facilitators and barriers associated with participation in lung cancer screening; Table S2: Quality assessment result of qualitative included studies; Table S3: Summary of the articles included in the review and primary finding of barriers and facilitators related to lung cancer screening (LCS) (n = 52).Table S4: Themes for facilitators to lung cancer screening. Table S5: Themes for barriers to lung cancer screening.

## Abbreviations

CASP	Critical Appraisal Skills Program
CMS	Centers for Medicare and Medicaid Services
ENTREQ	Enhancing Transparency in Reporting the Synthesis of Qualitative Research
LCS	Lung cancer screening
LDCT	Low-dose computerized tomography
MIPS	Merit-based incentive payment system
PRISMA	Preferred Reporting Items for Systematic Reviews and Meta-Analyses
SEM	Social Ecological Model
