# Supplementary material for: Facilitators and Barriers to Lung Cancer Screening during Long COVID: A Global Systematic Review and Meta-Study Synthesis of Qualitative Research
Source: Int J Environ Res Public Health. 2024 Apr 25;21(5):534. doi: 10.3390/ijerph21050534 (PMC11121223; doi:10.3390/ijerph21050534)
Supplement: Supplementary file 1 [file ijerph-21-00534-s001.zip › Table S1.pdf]

Table S1. Themes, according to facilitators and barriers associated with participation in lung cancer screening.

Theme	Factor
**Facilitator**	
Individual (patient)	▪ Knowledge [37, 44, 49]<br>▪ Awareness of early detection message [21, 32-34, 37, 41, 42, 44-46, 52]<br>▪ Interest to check for Lung Cancer [28, 32-34, 37, 41, 42, 44-48]<br>▪ High motivation to know the LCS results (seeking reassurance [32, 33, 41, 44, 48, 63]).<br>▪ Openness to be screened [42].<br>▪ Perceived risk of Lung Cancer [34, 41, 45, 47]<br>▪ High Perceived Benefits [30, 41-43]<br>▪ Motivation to quit smoking [32, 33, 37, 44]<br>▪ Who has a positive CT result and needs follow-up surveillance or treatment [30]<br>▪ Provision of the mobile testing program or home test kits during COVID-19 [8, 48, 75]<br>▪ Considered screening a waste of money [9].<br>▪ Online and telephone pre-screening decision-making and follow-up support [48]
Interpersonal (Provider-patient)	▪ Receiving a screening recommendation from a healthcare provider [21, 30, 33, 37, 44]<br>▪ Shared Decision-Making interaction between discussion [20, 31, 49, 50].<br>▪ Rapport with Care Coordinator rapport with the patient [20, 75]<br>▪ Good Doctor-patient relationships [42, 75]
▪ Social<br>▪ (cultural)	▪ The value of life and perceptions of age [40]<br>▪ Altruism [50, 52]
▪ Organizational (Institutional policy)	▪ A decision-making aid [50, 52]<br>▪ Reduced costs in specific facilities (V.A. and some Federally qualified) [30, 37, 48, 51, 52]<br>▪ Already established healthcare (VA setting) [16, 30, 51]
**Barriers**	
▪ Individual<br>▪ (patient)	▪ Low awareness of lung screening [28, 30-33, 35-38, 40, 43, 44, 46, 48, 53-55]<br>▪ Language or literacy problems [49]<br>▪ Insufficient input from the provider [50, 63]<br>▪ Self-efficacy [8, 20, 29, 44, 48, 62]<br>▪ Dislikes Hospital, healthcare system, Scans, and tests) [44]<br>▪ Patient's other Comorbidities [21, 51]<br>▪ Fear of cancer diagnosis and worry [9, 31-33, 35-37, 39, 42, 44-46, 50, 54, 56, 57].<br>▪ Low Perceived Benefit (Feeling healthy) [30, 36, 46, 58]<br>▪ Concern or High Perceived Risk of LDCT [28, 33, 34, 37, 40, 41, 43-45, 47, 56]<br>▪ Concern cancer itself [21, 34, 41, 47]<br>▪ Futility: denies or questions the utility of treatment or procedure [28, 44, 54],

	▪ Practical barrier [31-34, 37, 41, 45, 47, 48, 56]<br>▪ Smoking and cancer related stigma [9, 32-35, 39, 44]<br>▪ Being health conscious [45]<br>▪ lack of family(social) support to attend screening [36, 39, 48].<br>▪ COVID-19 fear (perceptions of risk, mortality, worry, behavioral and psychosocial responses to COVID-19 [8, 57, 59-61]<br>▪ Disruptions in cancer care by COVID-19 [57, 60, 76].
▪ Individual<br>▪ (provider)	▪ PCP inadequate Knowledge of LCS (Screening method, locations, eligibility criteria, and insurance coverage) [20, 29, 49, 51]<br>▪ Primary care's competing priorities [39, 50]<br>▪ Fear of causing misunderstanding during a risk-benefit conversation[46, 49].<br>▪ Discontinuity of care [75]<br>▪ Shared Decision [18, 20, 29, 49, 50]<br>▪ Inadequate dedicated time for SDM [18, 58]
▪ Interpersonal (Provider-patient)	▪ Lack of established relationship [18, 29]<br>▪ Poor rapport [18, 44, 63]<br>▪ Patient Education (provider recommendation) [21, 30, 36, 41, 45, 47, 53, 58, 62]<br>▪ False-Positive worry after discussion with the provider [44]<br>▪ The Decision Aid tool is confusing [50, 62].<br>▪ Patient Misunderstanding [35, 48, 58]<br>▪ Inadequate encounter time [29, 30, 49, 51, 63]
▪ Social (cultural)	▪ Distrust in the service/health professional [28, 31-34, 36, 56, 62]<br>▪ Fatalistic beliefs [28, 31, 33, 35, 41, 44, 46, 56]<br>▪ Perception of Aging [35, 36]
▪ Organizational (Institutional policy)	▪ Transportation [40, 63]<br>▪ Costs and copays [21, 28, 30, 36, 42, 51, 62]<br>▪ Lack of insurance coverage [20, 21, 37]<br>▪ Inadequate infrastructure [16, 21, 43, 51, 64]<br>▪ Lack of care coordinators [43, 52, 62],<br>▪ Communication between providers [21],<br>▪ Lack of institutional policy [16, 43, 50, 64],<br>▪ Long wait times [50].<br>▪ Public awareness [36]

Reference

1. Rolfo, C., et al., *Lung Cancer and SARS-CoV-2 infection: Identifying important knowledge gaps for investigation.* Journal of Thoracic Oncology: Official Publication of the International Association for the Study of Lung Cancer, 2021.
2. Ferlay, J., et al., *Cancer statistics for the year 2020: An overview.* International journal of cancer, 2021. **149**(4): p. 778-789.
3. *LUNG HEALTH, A Call to Action on Lung Cancer Screenings.* Future of personal health, 2023.
4. Tindle, H.A., et al., *Lifetime smoking history and risk of lung cancer: results from the Framingham Heart Study.* JNCI: Journal of the National Cancer Institute, 2018. **110**(11): p. 1201-1207.
5. Lin, Y.-a., et al., *Barriers and facilitators to uptake of lung cancer screening: A mixed methods systematic review.* Lung Cancer, 2022.
6. Cara Murez, H.N., *Cancer organizations push for increase in lung cancer screening.* NOV. 2, 2022 / 3:17 PM
7. Fuerst, M.L., *Data Shows Low-Dose CT Screening Reduces Lung Cancer Mortality*, 2021, LWW.
8. Brown, L.R., et al., *Increasing uptake to a lung cancer screening programme: building with communities through co-design.* BMC Public Health, 2022. **22**(1): p. 1-17.
9. Quaife, S.L., et al., *Smokers' interest in a lung cancer screening programme: a national survey in England.* BMC cancer, 2018. **18**(1): p. 1-10.
10. Lei, F., et al., *Health beliefs toward lung cancer screening among Chinese American high-risk smokers: Interviews based on Health Belief Model.* International journal of nursing sciences, 2022. **9**(3): p. 378-388.
11. Silvestri, G.A., et al., *Attitudes towards screening for lung cancer among smokers and their non-smoking counterparts.* Thorax, 2007. **62**(2): p. 126-130.
12. Weller, S.C., *Cancer and colorectal cancer: knowledge, beliefs, and screening preferences of a diverse patient population. Fam Med.* Fam Med, 2005. **37**(5): p. 341-7.
13. Margolis, M.L., et al., *Racial differences pertaining to a belief about lung cancer surgery: results of a multicenter survey.* Annals of internal medicine, 2003. **139**(7): p. 558-563.
14. Fang Lei, B. *Barriers to lung cancer screening with low-dose computed tomography*. in *Oncology Nursing Forum*. 2019. Oncology Nursing Society.
15. Cheng, Y.I., et al., *Implementation planning for lung cancer screening in China.* Precision Clinical Medicine, 2019. **2**(1): p. 13-44.
16. Klarenbeek, S.E., et al., *Barriers and facilitators for implementation of a computerized clinical decision support system in lung cancer multidisciplinary team meetings—a qualitative assessment.* Biology, 2020. **10**(1): p. 9.
17. Oudkerk, M., et al., *Lung cancer LDCT screening and mortality reduction—evidence, pitfalls and future perspectives.* Nature reviews Clinical oncology, 2021. **18**(3): p. 135-151.
18. Crothers, K., et al., *Patients' attitudes regarding lung cancer screening and decision aids. A survey and focus group study.* Annals of the American Thoracic Society, 2016. **13**(11): p. 1992-2001.
19. Richards, M., et al., *The impact of the COVID-19 pandemic on cancer care.* Nature Cancer, 2020. **1**(6): p. 565-567.
20. Hoffman, R.M., et al., *Peer Reviewed: Attitudes and Beliefs of Primary Care Providers in New Mexico About Lung Cancer Screening Using Low-Dose Computed Tomography.* Preventing chronic disease, 2015. **12**.
21. Holman, A., et al., *Patient perspectives on longitudinal adherence to lung cancer screening.* Chest, 2022. **162**(1): p. 230-241.
22. Moher, D., et al., *Preferred reporting items for systematic review and meta-analysis protocols (PRISMA-P) 2015 statement.* Systematic reviews, 2015. **4**(1): p. 1-9.
23. Tong, A., et al., *Enhancing transparency in reporting the synthesis of qualitative research: ENTREQ.* BMC medical research methodology, 2012. **12**(1): p. 1-8.

24. Paterson, B.L., et al., *Meta-study of qualitative health research: A practical guide to meta-analysis and meta-synthesis*. Vol. 3. 2001: Sage.
25. Beeker, C., et al., *Colorectal cancer screening in older men and women: qualitative research findings and implications for intervention.* Journal of community health, 2000. **25**(3): p. 263-278.
26. McLeroy, K.R., et al., *An ecological perspective on health promotion programs.* Health education quarterly, 1988. **15**(4): p. 351-377.
27. Janz, N.K. and M.H. Becker, *The health belief model: A decade later.* Health education quarterly, 1984. **11**(1): p. 1-47.
28. Gressard, L., et al., *A qualitative analysis of smokers' perceptions about lung cancer screening.* BMC Public Health, 2017. **17**(1): p. 1-8.
29. Melzer, A.C., et al., *What exactly is shared decision-making? A qualitative study of shared decision-making in lung cancer screening.* Journal of general internal medicine, 2020. **35**: p. 546-553.
30. Sin, M.-K., A. Ha, and V. Taylor, *Sociocultural barriers to lung cancer screening among Korean immigrant men.* Journal of community health, 2016. **41**: p. 790-797.
31. Carter-Harris, L., et al., *A qualitative study exploring why individuals opt out of lung cancer screening.* Family practice, 2017. **34**(2): p. 239-244.
32. Carter-Harris, L., et al., *Lung cancer screening: what do long-term smokers know and believe?* Health Expectations, 2017. **20**(1): p. 59-68.
33. Quaife, S.L., et al., *Attitudes towards lung cancer screening in socioeconomically deprived and heavy smoking communities: informing screening communication.* Health Expectations, 2017. **20**(4): p. 563-573.
34. Roth, J.A., et al., *A qualitative study exploring patient motivations for screening for lung cancer.* PLoS One, 2018. **13**(7): p. e0196758.
35. Tod, A.M., J. Craven, and P. Allmark, *Diagnostic delay in lung cancer: a qualitative study.* Journal of advanced nursing, 2008. **61**(3): p. 336-343.
36. Lobb, R., A.D. Pinto, and A. Lofters, *Using concept mapping in the knowledge-to-action process to compare stakeholder opinions on barriers to use of cancer screening among South Asians.* Implementation Science, 2013. **8**(1): p. 1-12.
37. Simmons, V.N., et al., *High-risk community and primary care providers knowledge about and barriers to low-dose computed topography lung cancer screening.* Lung Cancer, 2017. **106**: p. 42-49.
38. Cardarelli, R., et al., *Identifying community perspectives for a lung cancer screening awareness campaign in Appalachia Kentucky: the Terminate Lung Cancer (TLC) study.* Journal of Cancer Education, 2017. **32**: p. 125-134.
39. Kummer, S., et al., *Mapping the spectrum of psychological and behavioural responses to low-dose CT lung cancer screening offered within a Lung Health Check.* Health Expectations, 2020. **23**(2): p. 433-441.
40. Patel, D., et al., *Attitudes to participation in a lung cancer screening trial: a qualitative study.* Thorax, 2012. **67**(5): p. 418-425.
41. Tonge, J.E., et al., *"To know or not to know…?" Push and pull in ever smokers lung screening uptake decision-making intentions.* Health Expectations, 2019. **22**(2): p. 162-172.
42. Mishra, S.I., et al., *Peer reviewed: patient perspectives on low-dose computed tomography for lung cancer screening, New Mexico, 2014.* Preventing chronic disease, 2016. **13**.
43. Margariti, C., et al., *Healthcare professionals' perspectives on lung cancer screening in the UK: a qualitative study.* BJGP open, 2020. **4**(3).
44. Greene, P.A., et al., *Challenges to educating smokers about lung cancer screening: a qualitative study of decision making experiences in primary care.* Journal of Cancer Education, 2019. **34**: p. 1142-1149.
45. Draucker, C.B., et al., *Understanding the decision to screen for lung cancer or not: a qualitative analysis.* Health Expectations, 2019. **22**(6): p. 1314-1321.
46. Lowenstein, M., et al., *Real-world lung cancer screening decision-making: Barriers and facilitators.* Lung Cancer, 2019. **133**: p. 32-37.

47. Schiffelbein, J.E., et al., *Barriers, facilitators, and suggested interventions for lung cancer screening among a rural screening-eligible population.* Journal of Primary Care & Community Health, 2020. **11**: p. 2150132720930544.
48. Dunlop, K.L., et al., *Motivation is not enough: A qualitative study of lung cancer screening uptake in Australia to inform future implementation.* Plos one, 2022. **17**(9): p. e0275361.
49. Abubaker-Sharif, M., et al., *Primary care physician perceptions of shared decision making in lung cancer screening.* Journal of Cancer Education, 2020: p. 1-9.
50. Wiener, R.S., et al., *Patient and clinician perspectives on shared decision-making in early adopting lung cancer screening programs: a qualitative study.* Journal of general internal medicine, 2018. **33**: p. 1035-1042.
51. Mejia, M.C., et al., *Key informants' perspectives on implementing a comprehensive lung cancer screening program in a safety net healthcare system: leadership, successes, and barriers.* Journal of Cancer Education, 2021: p. 1-8.
52. Sayani, A., et al., *Advancing health equity in cancer care: the lived experiences of poverty and access to lung cancer screening.* Plos one, 2021. **16**(5): p. e0251264.
53. Williams, R.M., et al., *Lung cancer screening decisional needs among African American smokers of lower socioeconomic status.* Ethnicity & Health, 2022. **27**(3): p. 565-583.
54. Dhavale, P., A. Koparkar, and P. Fernandes, *Palliative care interventions from a social work perspective and the challenges faced by patients and caregivers during COVID-19.* Indian journal of palliative care, 2020. **26**(Suppl 1): p. S58.
55. Richman, I.B., T.V. Prasad, and C.P. Gross, *Lost to follow up?: A qualitative study of why some patients do not pursue lung cancer screening.* Preventive Medicine Reports, 2022. **29**: p. 101909.
56. Ali, N., et al., *Barriers to uptake among high-risk individuals declining participation in lung cancer screening: a mixed methods analysis of the UK Lung Cancer Screening (UKLS) trial.* BMJ open, 2015. **5**(7): p. e008254.
57. Schellekens, M.P. and M.L. van der Lee, *Loneliness and belonging: Exploring experiences with the COVID-19 pandemic in psycho-oncology.* Psycho-oncology, 2020. **29**(9): p. 1399.
58. Kanodra, N.M., et al., *Primary care provider and patient perspectives on lung cancer screening. A qualitative study.* Annals of the American Thoracic Society, 2016. **13**(11): p. 1977-1982.
59. Hyland, K.A. and H.S. Jim, *Behavioral and psychosocial responses of people receiving treatment for advanced lung cancer during the COVID-19 pandemic: A qualitative analysis.* Psycho-oncology, 2020. **29**(9): p. 1387.
60. Guven, D.C., et al., *Perspectives, knowledge, and fears of cancer patients about COVID-19.* Frontiers in oncology, 2020. **10**: p. 1553.
61. *Study reports drop in lung cancer screening, rise in malignancy rates during spring COVID-19 surge [news release]. American College of Surgeons; December 17, 2020. https://www.facs.org/media/press-releases/2020/lung-screening-121720. Accessed December 18, 2020.*
62. Herb, J., et al., *Barriers and facilitators to early-stage lung cancer care in the USA: a qualitative study.* Supportive Care in Cancer, 2023. **31**(1): p. 21.
63. Hall, S.a.E., et al., *Lung cancer: an exploration of patient and general practitioner perspectives on the realities of care in rural Western Australia.* Australian Journal of Rural Health, 2008. **16**(6): p. 355-362.
64. Walton, L., et al., *Patient perceptions of barriers to the early diagnosis of lung cancer and advice for health service improvement.* Family practice, 2013. **30**(4): p. 436-444.
65. Feldstein, A.C., et al., *Patient barriers to mammography identified during a reminder program.* Journal of women's health, 2011. **20**(3): p. 421-428.
66. Reynolds, L.M., I.P. Bissett, and N.S. Consedine, *Emotional predictors of bowel screening: the avoidance-promoting role of fear, embarrassment, and disgust.* BMC cancer, 2018. **18**: p. 1-9.
67. Cavers, D., et al., *Development of an evidence-based brief 'talking'intervention for non-responders to bowel screening for use in primary care: stakeholder interviews.* BMC Family Practice, 2018. **19**(1): p. 1-10.
68. Erkmen, C.P., et al., *Adherence to annual lung cancer screening with low-dose CT scan in a diverse population.* Cancer Causes & Control, 2021. **32**: p. 291-298.

69. Payton, E., et al., *Parents' expectations of high schools in firearm violence prevention.* Journal of community health, 2017. **42**: p. 1118-1126.
70. Sharf, B.F., L.A. Stelljes, and H.S. Gordon, *'A little bitty spot and I'm a big man': patients' perspectives on refusing diagnosis or treatment for lung cancer.* Psycho-oncology, 2005. **14**(8): p. 636-646.
71. Powell, W., et al., *Medical mistrust, racism, and delays in preventive health screening among African-American men.* Behavioral Medicine, 2019. **45**(2): p. 102-117.
72. Carter-Harris, L. and M.K. Gould, *Multilevel barriers to the successful implementation of lung cancer screening: why does it have to be so hard?* Annals of the American Thoracic Society, 2017. **14**(8): p. 1261-1265.
73. Finlayson, K.W. and A. Dixon, *Qualitative meta-synthesis: a guide for the novice.* Nurse researcher, 2008. **15**(2).
74. Shaheen, M.Y., *AI in Healthcare: medical and socio-economic benefits and challenges.* ScienceOpen Preprints, 2021.
