# Supplementary material for: Facilitators and Barriers to Lung Cancer Screening during Long COVID: A Global Systematic Review and Meta-Study Synthesis of Qualitative Research
Source: Int J Environ Res Public Health. 2024 Apr 25;21(5):534. doi: 10.3390/ijerph21050534 (PMC11121223; doi:10.3390/ijerph21050534)
Supplement: Supplementary file 1 [file ijerph-21-00534-s001.zip › Tables S2-S5.pdf]

Table S2. Quality assessment result of qualitative included studies.

Quality assessment question	Abubaker et al. 2020[1]	Carter-Harris et.al. 2017[3]	Crothers et al.2016[4]	Gressard et al. 2017[5]	Hoffman et al. 2015[6]	Melzer et al. 2020[7]	Mejia et al2020[8]	Mishra et al.2016[9]	Mo-Kyung et al. 2016[10]	Schiffelbein et al.2020[11]
Was there a clear statement of the aim of the research?	✓	✓	✓	✓	✓	✓	✓	✓	✓	✓
Is a qualitative methodology appropriate?	✓	✓	✓	✓	✓	✓	✓	✓	✓	✓
Was the research design appropriate to address the aim of the research?	✓	✓	✓	✓	✓	✓	✓	✓	✓	✓
Was the requirement strategy appropriate to the aim of the research?	✓	✓	✓	✓	✓	✓	✓	✓	✓	✓
Was the data collected in a way that addressed the research issue?	✓	✓	✓	✓	✓	✓	✓	✓	✓	✓
Has the relationship between research and participant been considered?	X	X	✓	X	X	X	X	X	X	X
Have ethical issue been taken into consideration?	X	✓	X	✓	X	X	✓	X	X	X
Was the data analysis sufficiently rigorous?	✓	✓	✓	✓	✓	✓	✓	✓	✓	✓
Is there a clear statement of findings	✓	✓	✓	✓	✓	✓	✓	✓	✓	✓
How valuable is the research	✓	✓	✓	✓	✓	✓	✓	✓	✓	✓
✓ = Yes ; X=No;	Sharf et al. 2005[12]	Wiener et al[13]. 2018	Williams et al. 2020[14]	Ali, Noor, et al. 2015[15]	CarterHarris, Lisa, et al[3].	Lisa C- etal 2017 [16].	Preston etal2018[17]	Claire etal[18]	Lowenstein 2019[19],	Quaife et al. 2017[20],
Was there a clear statement of the aim of the research?	✓	✓	✓	✓	✓	✓	✓	✓	✓	✓
Is a qualitative methodology appropriate?	✓	✓	✓	✓	✓	✓	✓	✓	✓	✓
Was the research design appropriate to address the aim of the research?	✓	✓	✓	✓	✓	✓	✓	✓	✓	✓
Was the requirement strategy appropriate to the aim of the research?	✓	X	X	✓	✓	✓	✓	✓	✓	✓
Was the data collected in a way that addressed the research issue?	✓	✓	✓	✓	✓	✓	✓	✓	✓	✓
Has the relationship between research and participant been considered?	✓	X	X	✓	X	✓	✓	X	X	X

Have ethical issue been taken into consideration?	X	✓	X	✓	✓	✓	✓	✓	X	✓
Was the data analysis sufficiently rigorous?	✓	✓	✓	✓	✓	✓	✓	✓	✓	✓
Is there a clear statement of findings	✓	✓	✓	✓	✓	✓	✓	✓	✓	✓
How valuable is the research	✓	✓	✓	✓	✓	✓	✓	✓	✓	✓
= Yes ; X=No;	32 Lynsey Rachael Brown, etal . 2022[2]	Aislinn Antrim 2020	Jennifer M, 2021	Schell kens, 2020	Guven at al, 2020	Hyland 2020	Dhaval 2020	Kate L. A. Dunlop etal	Simmons et al. 2017, Lung Cancer	Tonge et al. 2019, Health Expectations
Was there a clear statement of the aim of the research?	✓	✓	X	✓	✓	✓	✓	✓	✓	✓
Is a qualitative methodology appropriate?	✓	X	X	✓	✓	✓	✓	✓	✓	✓
Was the research design appropriate to address the aim of the research?	✓	X	X	✓	✓	✓	✓	✓	✓	✓
Was the requirement strategy appropriate to the aim of the research?	X	✓	X	✓	X	✓	X	✓	x	✓
Was the data collected in a way that addressed the research issue?	✓	X	X	✓	✓	✓	✓	✓	✓	✓
Has the relationship between research and participant been considered?	X	X	X	x	X	X	X	X	X	X
Have ethical issue been taken into consideration?	✓	X	✓	✓	✓	✓	✓	✓	✓	✓
Was the data analysis sufficiently rigorous?	✓	X	X	✓	✓	✓	✓	✓	✓	✓
Is there a clear statement of findings	✓	X	X	✓	✓	✓	X	✓	✓	✓
How valuable is the research	✓	✓	✓	✓	✓	✓	✓	✓	✓	✓
= Yes ; X=No;	Sayani, Ambreen, et al. 2021	Walton, Lisa, et al.2013	Hall, Son? A. E., et al.	Angela M et al.	Dunlop, Kate LA, et al, 2022	Ilana B. Richman et al. 2022	Holman, Anna, et al. 2022	Kanodra, Neeti M., et al. 2016	Klarenbeek, Sosse E., et al.	Quaife, Samantha L., et al.
Was there a clear statement of the aim of the research?	✓	✓	✓	✓	✓	✓	✓	✓	✓	✓
Is a qualitative methodology appropriate?	✓	✓	✓	✓	✓	✓	✓	✓	✓	✓
Was the research design appropriate to address the aim of the research?	✓	✓	✓	✓	✓	✓	✓	✓	✓	✓

Was the requirement strategy appropriate to the aim of the research?	✓	✓	X	✓	✓	✓	X	✓	✓	✓
Was the data collected in a way that addressed the research issue?	✓	✓	✓	✓	✓	✓	✓	✓	✓	✓
Has the relationship between research and participant been considered?	X	X	X	X	X	X	X	X	X	X
Have ethical issue been taken into consideration?	✓	✓	✓	✓	✓	✓	X	✓	✓	✓
Was the data analysis sufficiently rigorous?	✓	✓	✓	✓	✓	✓	✓	✓	✓	✓
Is there a clear statement of findings	X	X	X	✓	✓	✓	✓	✓	✓	✓
How valuable is the research	✓	✓	✓	✓	✓	✓	✓	✓	✓	✓
= Yes ; X=No;	Herb,et al, 2023. [3]	Patel, D.et al 2012	Rodríguez-Rabassa, et al,2020	Sonja Kumar et al,2020	Roberto Cardarelli et al, 2015	Vani N. Simmons et.al	Rebecca Lobb et al 2013	Margariti, Charikleia, etal.2020	Hanna, Karim, et al. 2022	Sayani, Ambreen, et al. 2021
Was there a clear statement of the aim of the research?	✓	✓	✓	✓	✓	✓	✓	✓	✓	✓
Is a qualitative methodology appropriate?	✓	✓	✓	✓	✓	✓	✓	✓	✓	✓
Was the research design appropriate to address the aim of the research?	✓	✓	✓	✓	✓	X	✓	✓	✓	✓
Was the requirement strategy appropriate to the aim of the research?	✓	✓	✓	✓	✓	✓	✓	X	✓	✓
Was the data collected in a way that addressed the research issue?	✓	✓	✓	✓	✓	✓	✓	✓	✓	✓
Has the relationship between research and participant been considered?	x	X	X	✓	X	X	X	X	X	X
Have ethical issue been taken into consideration?	✓	✓	✓	✓	X	X	✓	✓	✓	✓
Was the data analysis sufficiently rigorous?	✓	✓	✓	✓	✓	✓	✓	✓	✓	✓
Is there a clear statement of findings	✓	✓	✓	✓	✓	✓	✓	✓	✓	✓
How valuable is the research	✓	✓	✓	✓	X	✓	✓	✓	✓	✓
= Yes; X=No;										

Table S3. Summary of the articles included in the review and primary finding of barriers and facilitators related to lung cancer screening (LCS) (n = 52).

No	Author and year	Country	Study aim	Study design and Sample	Primary findings	Study conclusion and implication
1	Abubaker-Sharif et al. 2020[1]	USA	Provider perceptions of shared decision making in LCS	**Design**<br>-Semi-structured interviews and thematic analysis.<br>***Participants***<br>Primary care providers<br>***Sample size***<br>N=16<br>**Setting**<br>Via Email<br>**Gender**<br>Female (50%)<br>Male (50%)<br>**Age**<br>None	**Facilitators**<br>✓ Health providers' and patients' knowledge; shared decision-making implementation and practice.<br>**Barriers**<br>✓ Patients' fears and health literacy; LCS practice/decision support for providers; integrating decision counseling in practice.<br>**Other Factors**	**Conclusion**<br>▪ Primary Care Physician perception plays an important role in disseminating information about LCS and understanding the importance of shared decision-making (SDM).<br>**Implication**<br>▪ At the organizational level more needs to be done to ensure providers are trained in shared decision-making (SDM) and there is time allotted for the practice.
2	Carter-Harris et. al. 2017[4].	USA	Exploring why long-term smokers opt out of lung cancer screening	**Design**<br>Semi-structured interview And thematic content analysis.<br>***Participants***<br>*Individuals*<br>***Sample size***<br>*N=18*<br>**Setting**<br>Telephone interviews.<br>**Gender**<br>Male = 7<br>Female = 11<br>**Age**<br>55-77	**Facilitators**<br>✓ Patient-provider discussion about LC and reasons for opting out of LCS (e.g., knowledge avoidance, perceived low value, false positive worry, and patient misunderstanding).<br>**Barriers**<br>✓ Distrust and stigma<br>**Other Factors**<br>None	**Conclusion**<br>▪ Distrust and stigma must be addressed as more people are eligible for LCS.<br>**Implication**<br>▪ Distrust/ stigma may hinder implementation efforts.<br>▪ Shared-decision- making process between providers and high-risk patients is key.
3	Crothers et al. 2016[5]	USA	Determine the perspectives of vulnerable patients' understanding and preference of LCS decision aids.	**Design**<br>Focus groups, surveys (pre/post) and thematic analysis.<br>***Participants***<br>*Individuals (patient)*<br>***Sample size***<br>45 patients	**Facilitators**<br>**Barriers**<br>✓ Time shortage<br>✓ Translating medical jargon to understandable terminology<br>✓ Difficult taking about harms, benefits, and result LCS.	**Conclusion**<br>- Engaging patients in shared decision-making, including talking about harms, benefits, and results of LCS, is difficult under ideal circumstances and is limited by appointment time constraints,

				**Setting**<br>Hospital<br>**Gender**<br>Man=71%<br>Women=29%<br>**Age**<br>Averaging 61 years old	✓ Difficult to engage patients in shared decision-making.<br>**Other factors**	translating medical jargon to understandable terminology, and other barriers.<br>**Implication**<br>▪ Discussing LCS within a vulnerable patient population may be uniquely challenging, but the participants in our study generally increased their understanding of harms and benefits after reviewing decision aids and discussing screening.<br>▪ This finding can inform further work in the implementation of lung cancer screening among similar populations.
**4**	**Gressard et al. 2017[6]**	USA	Describe smokers. perceptions around LCS.	**Design**<br>Focus group discussion and constant comparative analysis.<br>***Participants***<br>*individuals*<br>***Sample size***<br>N=12<br>**Setting**<br>Website on institute<br>**Gender**<br>Male and female<br>**Age**<br>41-67,	**Facilitator**<br>Many participants were. unaware of LCS tests and those that were screened did not remember. information regarding the test. Many expressed a desire for LCS.<br>**Barriers**<br>1) health care system level (cost of procedure, confusion around results),<br>2) cultural level (fatalistic beliefs, distrust of medical system),<br>3) individual level (lack of knowledge, denial of risk, concerns about the procedure).<br>**Other factors**	**Conclusion**<br>Need clear patience and friendly. educational tools to improve. patient understanding of screening. risks and benefits.<br>**Implication**<br>Implementation of lung cancer screening should include sufficient. education and tools to improve patient understanding of the benefits and risks of screening.
**5**	**Hoffman et al. 2015[7]**	Mxico	Describe attitudes. and beliefs of primary care providers re: LCS using LDCT.	**Design**<br>Focus group discussions.<br>***Participants***	**Facilitators**<br>**Barriers**	**Conclusion**<br>• Providers have several concerns about the feasibility and

				Individuals<br><br>***Sample size***<br>*N=45*<br>**Setting**<br><br>Hospital<br>**Age**<br>50–74 years<br>**Gender**<br>Male and female<br>.	✓ Inadequate knowledge of provider to interpret results, current guideline recommendations.<br>✓ Communication challenges with patience; skepticism of results.<br>✓ Low efficacy of infrastructure; providers' perspectives conflicting with the SDM discussion.<br>✓ complexity of discussing benefits and harms of screening and surveillance with their patient population.<br>✓ Financial burdens for rural,<br>✓ Underinsured populations.	appropriateness of implementing LDCT screening.<br>• Effective lung cancer screening programs will need to educate providers and patients to support informed decision making and to ensure that high-quality screening can be efficiently delivered in community practice.<br>**Implication**<br>- both providers and patients need to be educated.<br>- organizational structures need to allow for shared.<br>- decision making process and infrastructure efficacy.
**6**	**Melzer et al. 2020[8]**	USA	Describe clinician perspectives.<br>on LCS and<br>their experience with the implementation process.	**Design**<br>Multicenter qualitative study<br>***Participants***<br>Health care provider and patient<br>***Sample size***<br>N=85<br>**Setting**<br>Hospital<br>**Age**<br>42<br>**Gender**<br>Male and female	**Facilitators**<br>**Barriers**<br>✓ Lack of time led to lower priority for completion.<br>✓ Lack of patient engagement in the process of decision-making.<br>✓ Many patients were perceived as being.<br>✓ uninterested in details and terminated the discussion.<br>**Other factors**	**Conclusion**<br>The lack of completion of some elements, such as PCPs' lack of in-depth information exchange, may reflect perceived patient preferences for communication.<br>**Implication**<br>As LCS is implemented, further research is needed to support a personalized, patient centered approach to produce better outcomes.
**7**	**Mejia et al 2020[9]**	USA	Perceptions of adoption of screening and appropriate referral practices across 15 community health centers.	**Design**<br><br>Qualitative study with key informants<br>***Participants***<br>*Patient*	**Facilitators**<br>✓ Allocation of resources and<br>services coverage	**Conclusion**<br>Results may inform interventions, especially organizational- level support.<br>**Implication**

				Sample size<br>N=53<br>**Setting**<br>community health centers<br><br>**Age**<br>**Gender**	✓ Need for a collaborative process to engage stakeholders and identify champions.<br>**Barriers**<br>✓ Lack of resources for screening<br>✓ Treatment insufficient time to address.<br>✓ Complex patient problems<br>✓ perceived lack of patient buy-in	- Findings inform EBP implementation efforts regarding resources and key barriers to success around organizational level supports and promotion of suitable EBP programs.<br>✓ Stakeholders need to be different types of evidence to support implementation.
8	**Mishra et al. 2016[10]**	USA	Describe patient perspectives on LDCT for LCS	**Design**<br>semi structured qualitative interviews<br>***Participants***<br>patients<br>***Sample size***<br>N=22<br>**Setting**<br>Health facility<br>**Age**<br>50-80<br>**Gender**<br>Male and Female	**Facilitators**<br>✓ Most Chest Clinic participants were unaware of LDCT screening.<br>✓ They were particularly mindful about costs but said they would make sacrifices to obtain necessary care.<br>✓ quality communication between patient-providers, decision-making aids.<br>**Barriers**<br>✓ Barriers to LCS were costs,<br>✓ Fear of radiation exposure, and transpiration<br>✓ psychological distress (stress, anxiety)<br>**Other factors**	**Conclusion**<br>▪ Participation in LCS among sociodemographic ally diverse patients require shared decision-making process and decision aids designed for People with low literacy.<br>**Implication**<br>• Implementing lung cancer screening in socio demographically diverse populations poses significant challenges.<br>• The value of tobacco cessation counseling cannot be overemphasized.
9	**Mo-Kyung Sin et al. 2016[11]**	USA	Explore barriers and facilitators to LCS among Korean immigrants<br>men	**Design**<br>Focused group and individual interview<br>***Participants***<br>*individuals Immigrants*<br>***Sample size***<br>N=24	**Facilitators**<br>✓ participants believed a good health care system, and that health care is cheaper and more convenient in Korea than in the US.<br>✓ receive low dose CT of the chest as part of preventive care	**Conclusion**<br>**Implication**<br>The findings used to design culturally and linguistically appropriate community-based lung cancer screening intervention programs for men of Korean descent, as well

				**Setting**<br>Churches and senior centers in the greater Seattle area.<br>**Age**<br>55-79<br>**Gender**<br>Men	package offered by the medical tourism industry.<br>✓ This will potentiality be problematic for men who have positive CT result and need follow up surveillance or treatment.<br>**Barriers**<br>✓ cost of health care<br>✓ Lack of time<br>✓ Lack knowledge.<br>✓ Lack of physician recommendation<br>✓ Attitude about prevention<br>**Other factors**	as intervention programs for health care providers who serve them.
**10**	**Schiffelbein et al. 2020[12]**	USA	Identify barriers/facilitators.<br>to LCS and interventions in a rural population.	**Design**<br>Qualitative and quantitative data with focus groups and completed a survey.<br>***Participants***<br>Rural residing adults<br>***Sample size***<br>N=23<br>**Setting**<br>**Age**<br>**Gender**	**Facilitators**<br>✓ Receiving a screening recommendation from a healthcare provider<br>✓ High motivation to know the screening results.<br>✓ Facilitator of annual screening:<br>✓ Receiving a true-positive result<br>**Barriers**<br>✓ Lack of knowledge regarding LCS, including screening method, locations, eligibility criteria, and insurance coverage<br>✓ Limited information or recommendations from providers,<br>✓ Lack of transportation.<br>✓ Feeling healthy (no symptoms)<br>✓ Barrier to annual screening:<br>✓ Receiving a negative or false-positive screening result	**Conclusion**<br>▪ The rural screening-eligible population is generally receptive to LCS.<br>▪ Patient-level factors important to getting this population screened include knowledge, transportation, motivation to know their screening results, and receiving information or recommendation from a provider.<br>**Implication**<br>✓ Primary care and community health practitioners should consider conducting targeted patient and public education efforts to encourage those at highest risk for lung cancer to speak with their PCPs about LCS<br>✓ Education initiatives in rural communities should be coupled with information about where and how to access LCS.

					Other factors	
11	**Sharf et al. 2005[13]**	USA	Identify perspectives. on refusing diagnosis or treatment to LC	**Design**<br>In-depth interviews.<br><br>***Participants***<br>Patient<br>***Sample size***<br>N=9<br>**Setting**<br>Hospital<br>**Age**<br>48-80 years<br>**Gender**<br>males	**Facilitators**<br>**Barriers**<br>- not been well cared for by the specialists who had talked with them about diagnostic and treatment options, which may have affected their decisions to refuse.<br>✓ Self-efficacy, minimizing threat, fatalism.<br>✓ faith, and distrust of medical authority: explanations were often multi-dimensional.<br>✓ Refusal to participate in deaths and logistics.<br>✓ Physical discomfort<br>✓ Living with uncertainty<br><br>**Other factors**	**Conclusion**<br>Attention for health care system continuity.<br>✓ Respect for the full spectrum of patient decision making options.<br>✓ Increasing uncertainty in the face of treating mortality or uncertain therapeutic benefit.<br>**Implication**<br>✓ Improved communication with patients about the likelihood of devastating diagnosis and constrictive way of relating to patient who chose not to follow their advice.<br>✓ Increasing trust while delivering bad news<br>✓ understanding the source of resistance to recommendations<br>✓ Discussing palliative care.
12	**Wiener et al[14]. 2018**	USA	Describe patient-clinician. perspectives and barriers to Shared. Decision-Making (SDM) related to Early Adopting Lung Cancer Screening Programs	**Design**<br>Qualitative approach with semi-structured interviews and focus groups.<br>***Participants***<br>clinicians and patients<br>***Sample size***<br>36clinicians and 49 patients<br>**Setting**<br>Hospitals<br>**Age**<br>**Gender**	**Facilitators**<br>✓ Clinicians believed the rationale and gave some (often purposely limited) information about the trade-offs of lung cancer screening.<br>**Barriers**<br>✓ they had not been well cared for by the specialists who had talked with them about diagnostic and treatment options, which may have affected their decisions to refuse.<br>✓ Insufficient time<br>✓ Competing priorities	**Conclusion**<br>Due to multiple perceived barriers, patient-clinician conversations about lung cancer screening may fall short of guideline-recommended shared decision-making supported by a decision aid. Consequently, patients may be left uncertain about lung cancer screening's rationale, trade-offs, and process.<br>**Implication**

					✓ Difficulty accessing decision aids.<br>✓ Limited patient comprehension and anticipated.<br>✓ patient emotions<br>**Other factors**	
**13**	**Williams et al. 2020[15]**	USA	Identify +/- factors. specific to LCS via LDCT and develop. value statements about the screening test for future research with African Americans.	**Design**<br>cross-sectional survey<br>***Participants***<br>Participants had a median 20 pack-years smoking history.<br>***Sample size***<br>*N* = 119<br>**Setting**<br>**Age**<br>55-80 years<br>**Gender**	**Facilitators**<br>**Barriers**<br>✓ They had not been well cared for by the specialists who had talked with them about diagnostic and treatment options, which may have affected their decisions to refuse.<br>✓ Most participants had not heard of LDCT and the total lung cancer screening knowledge.<br>**Other factors**	**Conclusions:**<br>Findings have implications for addressing the decisional needs of lower socioeconomic African American current and former smokers to promote informed decision-making for LDCT.<br>**Implication**
**14**	Ali, Noor, et al. 2015[16]	UK	to identify the barriers to participation among high-risk individuals in the UK Lung Cancer Screening (UKLS) pilot trial.	**Design**<br>Qualitative approach with Individuals. Data: semi-structured interviews. ***Participants***<br>High-risk individuals aged 50–75 years were invited to participate in UKLS<br>***Sample size***<br>*N* = 6817<br>**Setting**<br>Cambridge and Liverpool areas<br>**Age**<br>50–75 years<br>**Gender**	**Facilitators**<br>**Barriers**<br>✓ Age<br>✓ Gender<br>✓ Smoking status<br>✓ Socioeconomic group<br>✓ Travel with difficulties relating to the distance of travel.<br>✓ Lack of public transport available<br>✓ The cost of either the journey itself or hospital parking.<br>✓ avoidance of lung cancer information and fear<br>**Other factors**	**Conclusions:**<br>A profile of risk factors for nonparticipation in lung screening has emerged, with underlying reasons largely relating to practical and emotional barriers.<br>**Implication**<br>- Strategies for engaging high-risk, hard-to-reach groups are critical for the equitable uptake of a potential future lung cancer screening programmed.<br>- In the case of a national lung cancer screening programmed, efforts to improve uptake should include strategies for engaging women and those most at risk, including adults over 70 years, smokers, and those from deprived areas.

						- Practical barriers relating to access should be addressed, with behavioral interventions designed to minimize emotional barriers, especially among current smokers.
**15**	**Carter-Harris, Lisa**, et al[4].	Washington State	to explore the reasons for screening-eligible patients' decisions to opt out of screening after receiving a provider recommendation.	**<u>Design</u>**<br>Qualitative approach with Semi-structured telephone interviews<br>***<u>Participants</u>***<br>participants who met lung cancer screening criteria for age, smoking and pack-year history<br>***<u>Sample size</u>***<br>*N* = 18<br>**<u>Setting</u>**<br>Washington State<br>**<u>Age</u>**<br>55–74 years<br>**<u>Gender</u>**<br>Male and Female	**<u>Facilitators</u>**<br>✓ Patient never heard of lung cancer screening,<br>✓ The expectation of making an informed decision is a challenge.<br>**<u>Barriers</u>**<br>✓ Knowledge Avoidance<br>✓ Perceived Low Value<br>✓ False Positive Worry<br>✓ Practical Barriers<br>✓ Patient misunderstanding	**<u>Conclusions:</u>**<br>The participants in our study provided insights into why some patients make the decision to opt out of low dose computed tomography screening, which provides knowledge that can inform intervention development to enhance shared decision-making processes between long-term smokers and their providers and decrease decisional conflict about screening.<br>**<u>Implication</u>**<br>▪ With lung, patients and providers are new to both the screening option and the shared decision-making process adding layers of complexity to the Implementation of lung cancer screening.<br>▪ it is critically important that both patients and providers are supported in methods that foster a shared decision-making process.
**16**	**Lisa Carter-Harris etal** 2017 [17].	USA	To explore knowledge and beliefs of long-term smokers about lung cancer, associated risk factors and lung cancer screening.	**<u>Design</u>**<br>Qualitative study, four FGD with individuals. Analysis. Content analysis<br>***<u>Participants</u>***<br>Screened and unscreened long-term smokers.<br>***<u>Sample size</u>***	**<u>Facilitators</u>**<br>✓ Perceived benefits of screening identified include.<br>✓ Finding lung cancer early<br>✓ Giving peace of mind; and	**<u>Conclusions:</u>**<br>Perceived barriers to lung cancer screening, such as distrust and stigma, must be addressed as lung cancer screening becomes more widely implemented.<br>**<u>Implication</u>**

				N = 26<br>**Setting**<br>Central Indiana area<br>**Age**<br>Mean age of 66 years<br>**Gender**<br>Male and Female	✓ motivation to quit smoking.<br>**Barriers**<br>Perceived barriers to screening identified include:<br>✓ Inconvenience<br>✓ Distrust; and<br>✓ Stigma.	✓ Heightened levels of health-care system distrust may impact successful implementation of screening programmers.<br>✓ Perceived smoking-related stigma may lead to low levels of patient engagement with medical care and decreased cancer screening participation.<br>✓ It is also important to determine modifiable targets for intervention to enhance the shared decision-making process between health-care providers and their high-risk patients.
**17**	Preston A. Greene etal 2018[18]	USA	to identify current smokers' barriers to informed decision-making about LCS when it is offered during a routine primary care visit	**Design**<br>Qualitative, telephone semi-structured interviews with individuals<br>***Participants***<br>Smoking history of 49 pack-years.<br>***Sample size***<br>N=37<br>**Setting**<br>Lung Cancer Screening Clinical Demonstration Project (LCSCDP))<br>**Age**<br>55–74 **years**<br>**Gender**<br>Male	**Facilitators**<br>**Barriers**<br>✓ Fear of Lung Cancer<br>✓ Shame, Self-Blame, and Futility<br>✓ Perceived Ability of LCS to Quantify and Measure Risks and Harms<br>✓ Deference to Providers<br>✓ Lack of Interest and Understanding of Numerical Risk Information<br>✓ unique cognitive<br>✓ emotional<br>✓ social factors	**Conclusion**<br>**-** Participants appeared to poorly understand the benefits and risks of LCS. Most participants held very positive views of LCS and overwhelmingly regarded the perceived benefits of early detection as a compelling reason to participate in screening:<br>**Implication**<br>- Physicians may want to consider different ways to involve smokers in an explicit shared decision-making process, though it is notable that all elements generally considered essential for informed decision-making were not found in patients' accounts of the offer of LCS and subsequent decisions.
**18**	Claire Burke Draucker etal[19]	USA	To describe how current and former long-term smokers explain their	**Design**<br>Qualitative, telephone semi-structured interviews ***Participants***<br>Screened and not screened persons.	**Facilitators**<br>▪ Inaccurate beliefs that influenced their decisions about lung cancer screening participation.	**Conclusion**<br>▪ Education initiatives aimed at providers and long-term

			deci- sions regarding participation in lung cancer screening.	***Sample size***<br>N=40<br>**Setting**<br>United States<br>**Age**<br>55 to 70 years<br>**Gender**<br>Women and men	**Barriers**<br>✓ No intention to be screened,<br>✓ No deliberate consideration but somewhat open to being screened.<br>✓ Deliberate consideration but no definitive decision to be screened.<br>Intention to be screened and had been screened.	smokers regarding LDCT is needed.<br>▪ Quality patient/provider communication is most likely to improve screening rates.<br>**Implication**<br>- High-quality patient-provider communication about lung cancer screening should occur regularly.<br>- evolving, opportunities to discuss their hesitancy to screen with a provider would likely improve screening rates.
**19**	Lowenstein 2019[20], Lung Cancer	USA	To explore 1) attitudes and priorities among physicians and patients that inform shared decision-making about lung cancer screening in real-world settings and 2) physician and patient perceptions of shared decision-making in real-world lung cancer screening (LCS) practice.	**Design**<br>Qualitative interview ***Participants***<br>individuals and providers ***Sample size***<br>N=42<br>**Setting**<br>Primary care physicians (PCPs) in California.<br>**Age**<br>**Gender**	**Facilitators**<br>Physicians offered LCS inconsistently and were ambivalent about screening because of potential harms<br>✓ false positive results<br>✓ incidental findings<br>✓ radiation exposure.<br>**Barriers**<br>Perceived patient barriers<br>✓ Concern about cost<br>✓ Inadequate understanding of lung cancer risk<br>✓ Competing health issues<br>✓ Fear of a cancer diagnosis<br>✓ Fear of radiation<br>Physician barriers<br>✓ Complexity of the visit<br>✓ Lack of time during the visit<br>✓ Concern about false positives<br>✓ Confusion about the low-dose CT guidelines	**Conclusion**<br>▪ Physicians and patients expressed different concerns about LCS and different perceptions about the use of shared decision-making.<br>**Implication**<br>▪ Findings from this real-world population of screening-eligible patients can be used to inform the design of future interventions to facilitate communication and decision-making tailored to perspectives of both physicians and patients.

					✓ Confusion about referral pathways	
**20**	Quaife et al. 2017[21], Health Expectations	UK	To compare smokers' beliefs about lung cancer screening with those of former and never smokers within a low socioeconomic status (SES) sample, to explore the views of lower SES smokers and ex- smokers in- depth, and to provide insights into effective engagement strategies.	**Design**<br>Mixed methods, and thematic analysis.<br>**Sample size**<br>(N=184).<br>**Setting**<br>**Participants**<br>**Age**<br>**Gender**	**Facilitators**<br>**Barriers**<br>- Fatalism,<br>- worry and perceived risk of lung cancer<br>- Perceived blame and stigma around lung cancer	**Conclusion**<br>Attitudes towards screening among this high- risk group are complex.<br>**Implication**<br>Invitation strategies need to be carefully devised to achieve equitable participation in screening
**21**	Roth et al. 2018, PLoS One [22].	USA	To explore the motivations for screening-eligible patients to screen for lung cancer.	**Design**<br>in-depth interviews and inductive content analysis<br>**Participants**<br>with individuals<br>**Sample size**<br>(n=20).<br>**Setting**<br>**Age**<br>**=>55**<br>**Gender**<br>Female= 60<br>Male=40	**Facilitator**<br>-perceived benefit of early-detection,<br>- absence of safety concerns,<br>- personal relationships.<br>**Barriers**	**Conclusion**<br>- Our findings provide new insights about patient motivations to screen and can potentially be used to improve lung cancer screening uptake and shared decision-making processes.
**22**	Tonge et al. 2019, Health Expectations	UK	To explore with ever smokers the acceptability of targeted lung screening and uptake decision-making intentions	**Design**<br>Semi-structured focus group and thematic analysis.<br>**Participants**<br>With individuals<br>**Sample size**<br>N=94<br>**Setting**<br>**Age**<br>**Gender**	**Facilitators**<br>**Barriers**<br>-worry about diagnosis and screening tests.<br>- practicalities such as accessibility-<br>- perceptions of individual risk<br>- stigma.	**Conclusion**<br>Decision making was multifaceted with indications that current smokers faced higher participation barriers than ex-smokers.<br>**Implication**<br>Reducing participation barriers through careful service design and provision of decision support information will be important in

						lung screening programs to support informed consent and equitable uptake.
**23**	Simmons et al. 2017, Lung Cancer [	USA	to examine knowledge and attitudes about LDCT screening for lung cancer among an ethnically and racially diverse sample of high risk (HR) community members and primary care providers (PCP). -	**Design**<br>focus groups discussion and constant comparative method.<br>**Participants**<br>with individuals and providers<br>**Sample size**<br>(N=61).<br>**Setting**<br>**either in-patient or on home care receiving palliative care**<br>**Age**<br>- **18-67**<br>**Gender**<br>- Male and female	**Facilitator**<br>- Lack of information about LCS<br>- Never had a health care provider recommendation.<br>-<br>**Barriers**<br>- limited knowledge of lung cancer CT screening.<br>-Financial cost/insurance<br>- potential for false positives.<br>- fear of results (bad news)	**Conclusion**<br>Understanding the barriers to lung screening across diverse community populations is necessary to improve screening rates and shared decision-making.<br>**Implication**<br>---------
**24**	**Kate L. A. Dunlop etal[22].**	Australia	to identify the potential drivers of participation in LCS in the Australian setting, to inform future implementation.	**Design**<br>Semi-structured interview and using the COM-B model analysis.<br>**Participants**<br>interviews with individuals<br>**Setting**<br>telephone<br>**Sample size**<br>(n=39).<br>Age<br>gender	**Facilitators**<br>low self-efficacy.<br>-in capability including ability to attend and in knowledge and understanding.<br>**Barriers**<br>- challenges related to physical and social opportunity<br>- lack of family support to attend screening.	**Implication**<br>▪ Focusing strategies on barriers related to capability and opportunity such as online/telephone support, mobile screening programs and financial assistance for screeners may better enhance screening participation.<br>▪ Providing funding for clinicians to support individuals in decision making and belief in self-efficacy may foster motivation.<br>▪ Targeting interventions that connect eligible individuals with the LCS program will be crucial for successful implementation.
**25**	Dhaval 2020[23]	India	To describe the challenges faced by patients and caregivers during the lockdown due to the COVID-19 pandemic.	**Design**<br>A qualitative study with framework analysis<br>**Participants**<br>individuals	**Facilitators**<br>**Barriers**<br>- physical distress due lack of availability of medicines and nursing care	-

				**Sample size**<br>(N=30).<br>**Setting**<br>**Gender**<br>**Age**	-emotional distress due to the interruption of cancer treatment<br>- financial and social distress about loss of incomes<br>- isolation and spiritual distress due to the uncertainty of last rites as well as fulfilment of last wishes.<br>- lack of information about available services and confidence to ask for help from others as well as dealing with the grief of a dying relative.	
**26**	Hyland 2020[24]	USA	To characterize the behavioral and psychosocial responses of people with advanced lung cancer to the COVID-19 pandemic.	**Design**<br>Semi-structured interview and them analysis<br>**Participants**<br>with individuals<br>**Sample size**<br>(N=15)<br>**Setting**<br>Via mail and telephone<br>**Gender**<br>**Age**	**Facilitators**<br>**Barriers**<br>- Awareness of mortality<br>- perceptions. of risk,<br>- behavioral and psychosocial responses to COVID-19,<br>- sense of loss/ mourning and positive reinterpretation/greater appreciation of life.	**Conclusion**<br>All participants reported changing their behavior in response to COVID-19.<br>**Implication**<br>Findings provide important and novel insight into patients' perceptions of and experience during COVID19 and have implications for oncology providers.
**27**	Guven at al[25], 2020	Turkey	To assess the perspectives and fears of cancer patients about COVID-19.	**Design**<br>**Participants**<br>individuals<br>**Sample size**<br>(n=250)<br>**Setting**<br>online<br>**Gender**<br>**Age**	**Facilitators**<br>**Barriers**<br>- COVID-19 fear.<br>- disruptions in cancer care by Covid-19.	**Conclusion**<br>A significant amount of our patients had the wrong information about protection necessities and discontent about the adequacy of information, which denote the need for better patient education about COVID-19.
**28**	Schell kens[26] 2020	Netherlands	To explore experiences with the COVID-19 pandemic in patients or family members who sought help at a mental	**Design**<br>**Participants**<br>with individuals<br>**Sample size**<br>(n=871)	**Facilitators**<br>- **communication between clients and health care professionals.**<br>**Barriers**	**Implication**<br>- When fear and feelings of loneliness are addressed and normalized, it often relieves accompanying distress.

			healthcare institute for psycho-oncology.	**Setting**<br>online<br>**Gender**<br>**Age**<br>**30-70**	- patients feared not being able to say farewell to family and friends in case of dying from COVID-19.<br>- feeling lonely which stimulated their worries regarding cancer.<br>- lockdown because Covid-19.	- Importantly, addressing these feelings might also help to take the steps to seek psychological care if needed.
**30**	Jennifer M[28]. 2021	USA	To assess Cancer screening through the COVID-19 pandemic, recovery, and beyond	**Design**<br>N/A<br>**Participants**<br>N/A<br>**Sample size**<br>N/A<br>**Setting**<br>N/A<br>**Gender**<br>N/A<br>**Age**<br>N/A	**Barriers**<br>▪ Disruptions to care due to the pandemic	**Conclusion**<br>▪ Successful delivery of services at all points in the process has been negatively affected by the pandemic.<br>**Implication**<br>▪ There is a void in empirical high-quality evidence to support a specific strategy for administering cancer screening during a pandemic and its resolution phase, but several pragmatic considerations can help guide prioritization efforts.<br>▪ Targeting guideline-eligible people who have never been screened, or those who are significantly out of date with screening, has the potential to maximize benefits now and into the future.
**31**	Aislinn Antrim 2020	USA	To assess lung cancer screening and malignancy during Covid-19 Pandemic.	**Design**<br>**Participants**<br>Interview with providers<br>**Sample size**<br>**N/A**<br>**Setting**<br>Face to face<br>**Gender**<br>N/A<br>**Age**<br>**N/A**	**Facilitators**<br>**Barriers**<br>- patient fears about entering health facilities during the pandemic.	**Conclusion**<br>**Implication**<br>- we made a big emphasis within our program and with our nurses and coordinators to educate patients about those changes and to really get the message out that screening is safe.”

**32**	Lynsey Rachael Brown, etal . 2022[2]	UK	to identify ways to further increase opportunity for uptake of a lung cancer screening program,	**Design**<br>Qualitative study with<br>**Participant**<br>individuals and providers<br>**Sample size**<br>≥ 55<br>**Setting**<br>**Gender**<br>**Age**<br>N=27	**Facilitator**<br>- Provision of home test kits<br>- TV was the preferred means of communicating about the LCS.<br>**Barriers**<br>- Fear<br>- Need assistance to home test, e.g., nurse, pharmacist, or friend.	**Conclusion**<br>**Implication**<br>▪ Embedding the service in communities, Effective communication, Overcoming barriers with options.<br>▪ Continuing the process to develop these solutions in a collaborative way helps to encourage the personalized approach to delivery that is likely to improve uptake amongst groups that could benefit most.
**33**	Quaife, Samantha L., et al.[29]	UK	examined interest in a national lung cancer screening programme and modifiable attitudinal factors that may affect participation by smokers.	**Design**<br>Mixed study and content analysis.<br>**Participants**<br>-Individuals<br>**Sample size**<br>N=15<br>**Setting**<br>Face to face<br>**Gender**<br>**Age**<br>50-70	**Facilitators**<br>- Participants considered screening a waste of money.<br>**Barriers**<br>- Stigma<br>- Anxiety	**Conclusion**<br>- To improve smokers' participation, care should be taken to communicate the survival benefits of early-stage diagnosis and minimize anxiety and stigma related to lung cancer risk.<br>**Implication**<br>- Strategies aimed at engaging smokers with screening should focus on improving perceptions of the curability of early-stage disease and addressing concerns about screening.<br>- Communication throughout the screening process needs to be sensitively devised so that it is mindful of the existing stigma around smoking, and the anxiety smokers may have about their increased risk of lung cancer.
**34**	Klarenbeek, Sosse E., et al.[30]	Netherland	-To identify barriers and facilitators for implementation for the use case of lung cancer; and	**Design**<br>-semi structured interview and thematic analysis.<br>**Participant**	**Facilitator**<br>▪ Implementation of the computerized clinical decision support systems were considered.	**Conclusion**<br>- Successful implementation was seen as dependent on the reliabil-

			-To provide actionable findings for an implementation strategy	Providers<br>**Setting**<br>**Sample size**<br>N=26<br>**Gender**<br>**Age**	- to be easy access to well-structured patient data<br>- the resulting reduction of multidisciplinary team meetings preparation time and of duration of multidisciplinary team meetings<br>**Barriers**<br>- incomplete or non-trustworthy output generated by the system.<br>- insufficient adaptability of the system to local and contextual needs.	ity and adaptability of the computerized clinical decision support systems and involvement of key users in the implementation process.<br>**Implication**<br>- Using a computerized clinical decision support system in lung cancer multidisciplinary team meetings was expected to increase efficiency of workflows.<br>- Inform further decision strategies for successful implementation.
**35**	Kanodra, Neeti M., et al.[31]2016	USA	To identify perceptions of and perspectives on lung cancer screening and implementation among PCPs and eligible veteran patients at high risk for lung cancer.	**Design**<br>Qualitative study and thematic analysis.<br>**Participant**<br>Individual and providers<br>**Setting**<br>-Via email<br>**Sample size**<br>N=41<br>**Gender**<br>Male100%<br>**Age**<br>55-76	**Facilitator**<br>**Barriers**<br>-lack of patient understanding<br>-Primary care participants time constraints for lack of appropriate counseling and shared decision making.	**Implication**<br>- efforts are needed to improve guideline knowledge and adherence among providers.<br>- System-level interventions are necessary to facilitate time and resources for shared decision making and smoking cessation counseling and treatment.<br>- Further research is needed to identify optimal strategies
**36**	Holman, Anna, et al[32].2022.	USA	To explore patients' perspectives on barriers and facilitators to adherence to annual LCS.	**Design**<br>Qualitative study and framework analysis.<br>**Participant**<br>Individuals<br>**Setting**<br>Face to face<br>**Sample size**<br>N=40	**Facilitator**<br>- patient remainder<br>- provider recommendation<br>**Barriers**<br>- Cost, insurance coverage, accessibility, and other medical conditions.<br>- competing health concerns,<br>- less provider communication.	**Conclusion**<br>- patient reminders, provider recommendations may improve long-term screening behavior, and a number of barriers to the screening process could be addressed through patient navigation.<br>**Implication**

				**Gender**<br>**Age**		
**37**	Ilana B. Richman et al. 2022[33]	USA	to understand barriers to screening among a specific but important population: patients who have been referred for screening, but who have not completed the test.	**Design**<br>Qualitative study<br>**Participants**<br>Individuals<br>**Sample size**<br>N=12<br>**Setting**<br>Via telephone<br>**Gender**<br>Male: Female<br>**Age**<br>Mean=65 yr.	**Facilitator**<br>**Barriers**<br>- lack of knowledge	**Conclusion**<br>**-** lack of knowledge about screening is an important barrier to use, as patients are unlikely to prioritize a test if they know little about it.<br>**Implementation**<br>- opportunity to improve on current practice by developing tools to provide basic, accessible information about lung cancer screening for patients who may benefit.
**38**	Dunlop, Kate LA, et al, 2022.[34]	Australia	to identify the potential drivers of participation in LCS in the Australian setting, to inform future implementation.	**Design**<br>Semi structured telephone interview and CIM-B model of behavior analysis.<br>**Participants**<br>Individuals<br>**Sample size**<br>N=39<br>**Setting**<br>Telephone<br>**Gender**<br>**Male and female**<br>**Age**<br>55–80 years	**Facilitator**<br>-Providing online and telephone pre-screening decision making and follow-up support,<br>-Mobile screening programs to improve accessibility,<br>- Funding for clinicians to support individuals in decision-making would contribute to increasing participation in LCS.<br>**Barriers**<br>- low self-efficacy.<br>-lack of knowledge and understanding.<br>-Challenges related to physical and social opportunity<br>-lack of family support to attend screening.	**Conclusion**<br>- high level of motivation to participate in LCS for individuals at high-risk of lung cancer participating in a trial, driven by the lived experience of lung cancer and a belief in the value of screening.<br>**Implication**<br>-Online/telephone support, mobile screening programs and financial assistance for screeners may better enhance screening participation.<br>-Providing funding for clinicians to support individuals in decision making and belief in self-efficacy may foster motivation.<br>-Targeting interventions that connect eligible individuals with the LCS program will be crucial for successful implementation.
**39**	Angela M et al. [35]	UK	To explore and explain delay, particular pre-diag-	**Design**<br>-qualitative study and framework analysis.	**Facilitators**<br>None<br>**Barriers**	**Conclusion**

			nostic delay, in lung cancer and to consider the implications for public education and nursing.	**Participants**<br>Individuals<br>**Sample size**<br>N=20<br>**Setting**<br>At home<br>**Gender**<br>Male and female<br>**Age**<br>47-81	-lack of knowledge<br>- fear.<br>-Blame and stigma<br>-cultural factors,<br>-non-standard patterns of healthcare utilization -underlying stoical attitudes.	-Public education campaigns can worsen can worsen health inequalities if mis interpreted.<br>**Implication**<br>-Lack of knowledge and awareness about lung cancer could be addressed by better education of the public.<br>-Social marketing is a way of developing education messages which tackle cultural influences on treatment-seeking delay.<br>-Nurses have a potential role in developing and disseminating those messages.
**40**	Hall, Son? A. E., et al.[36]	West Australia	To explores the barriers to quality care in rural areas as perceived by GPs and patients.	**Design**<br>Qualitative study<br>**Participant**<br>Individual and providers<br>**Sample size**<br>N= 70<br>**Setting**<br>-Via telephone and face to face<br>**Gender**<br>Male: Female<br>**Age**<br>55-71	**Facilitator**<br>- Rural patients desire more information and better communication between hospitals and GPs.<br>-concerns about late confirmation of diagnosis.<br>**Barriers**<br>- They experienced longer waits for specialist consultation and underwent less diagnostic testing.<br>- Rural GPs reported distance, time and availability of appointments.<br>- Concerns about late confirmation of diagnosis.	**Conclusion**<br>-Rural patients received a different care pattern from metropolitan patients and they had their GP raise concern about the equity and quality of their lung cancer care.<br>**Implication**
**41**	Walton, Lisa, et al.2013 [37]	Newzland	explore New Zealand service users' experiences of the pathway to lung cancer diagnosis, identify factors con-trib-	**Design**<br>Interview and focus group discussion and thematic analysis.<br>**Participants**<br>Individual<br>**Sample size**<br>N=39	**Facilitator**<br><br>**Barriers**<br>- System complexity,<br>- information systems and resourcing issues	**Conclusion**<br>- Reason for not screening is complex and multi-factorial.<br>**Implementation**<br>- community initiatives to educate and resource at-risk patients to seek help, supporting

			uting to delay and provide advice for service improvement.	**Setting**<br>Face to face<br>**Gender**<br>**Age**<br>48-84		- resourcing primary care to increase timely referral and implementing strategies to reduce system complexity for GPs and patients, and the employment of care coordinators.
**42**	Sayani, Ambreen, et al. 2021[38]	Canada	To explore Perspectives of family physicians towards access to lung cancer screening for individuals living with low income.	**Design**<br>Semi structure interview and theory-informed thematic analysis.<br>**Participants**<br>Individual and providers.<br>**Sample size**<br>N=11<br>**Setting**<br>Via email (Online)<br>**Gender**<br>**N/A**<br>**Age**<br>N/A	**Facilitators**<br>- the degree of social disadvantage that influences lung cancer risk and opportunities to access care.<br>- a mismatch between the complex health needs of low-income individuals and structure of health care appointments.<br>- the need for equity-oriented health care, illustrated by the neglect of structural origins of health risk and the benefits of a trauma-informed approach.<br>**Barriers**	**Conclusion**<br>-An equity-oriented and interdisciplinary team-based approach to care will be needed in order to improve access to LCS, and attention must be given to the upstream determinants of lung cancer in order to reduce lung cancer risk.<br>**Implication**<br>-The multiprong strategies that will be needed in order to improve equity in health outcomes.
**43**	Sayani, Ambreen, et al. 2021 [39]	Canada	To explore the lived experiences of poverty and access to lung cancer screening.	**Design**<br>Qualitative interview and theoretical thematic analysis.<br>**Participant**<br>Individuals<br>**Sample size**<br>N=8<br>**Setting**<br>In person at primary care site<br>**Gender**<br>Male=90%<br>Female=10%<br>**Age**<br>55-74	**Facilitator**<br>-Any Health promotion program that focuses on individual uptake.<br>-<br>**Barriers**	**Conclusion**<br>-underserved population will require multiprong interventions that work at the individual, system and structural level to reduce inequity in lung cancer risk and access to health care services such as cancer screening.<br>**Implication**<br>-Underserved populations will require multiprong interventions that work at the individual, system and structural level to reduce inequities in lung-cancer risk and access to healthcare services such as cancer screening.

						- Research needed in to lived experiences of patients can continue to provide meaningful insights in to what works and what's needed to enhance health promoting opportunities.
**44**	Hanna, Karim, et al. 2022[40]	USA	To identify cancer screening barriers and facilitators during the pandemic in rural and urban primary care practices,	**Design**<br>Qualitative study and using deductive and inductive coding (hybrid approach) in NVivo 12 Plus.<br>**Participants**<br>Providers<br>**Sample size**<br>N=42<br>**Setting**<br>In person<br>**Gender**<br>**Age**<br>------	**Facilitators**<br>-home-based testing,<br>-using telehealth,<br>- strong partnerships with referral sites.<br>**Barriers**<br>- policy-level (eg, elective procedure delays),<br>-organizational (eg, backlogs),<br>- individual (eg, patient cancellation).	**Conclusion**<br>- Primary care staff used innovative strategies during the pandemic to promote cancer screening.<br>**Implementation**<br>- Primary care staff used innovative strategies during the pandemic to promote cancer screening.<br>- -Unresolved challenges (eg, backlogs and inability to implement telehealth) disproportionately affected rural clinics.<br>- incentivizing patients and providers and expanding outreach encourage screening.
**45**	Margariti, Charikleia, etal.2020[41]	UK	To explore healthcare professionals' views and perspectives about lung cancer screening and their preparedness and willingness to be involved in its implementation.	**Design**<br>- Qualitative study and thematic analysis.<br>**Participants**<br>- providers<br>**Sample size**<br>-N=16<br>**Setting**<br>face to face or by telephone.<br>**Gender**<br>Male =63%<br>Female=37%<br>**Age**<br>30-69	**Facilitators**<br>-acknowledged the health benefits of screening<br>**Barriers**<br>- lack of awareness and understanding,<br>- uncertainty and concerns about the validity of screening,<br>- the potential impact on their patients and workload.	**Implication**<br>- Addressing these concerns by providing resources and effective and detailed guidelines for their use may lead to greater engagement and willingness to be involved in lung cancer screening.

**46**	Rebecca Lobb et al[42], 2013	South Asian	To examined barriers to use of evidence-based interventions to improve early detection of cancer among South Asians	**Design**<br>Braine storming session and multi-dimensional scaling cluster analysis.<br>**Participants**<br>Individuals and providers<br>**Sample size**<br>N=53<br>**Setting**<br>**-**<br>**Gender**<br>**-**<br>**Age**<br>-	**Facilitators**<br><br>**Barriers**<br>- patient's beliefs,<br>- fears,<br>- lack of social support.<br>- limited knowledge among residents.<br>- limited knowledge among physicians.<br>- Lack of health education programs.<br>- Ethno-cultural discordance with the health system; and cost.	**Conclusion**<br>-- The limited reach of cancer control programs to racial and ethnic minority groups is a critical implementation issue that requires attention.<br>**Implication**<br>-Opinions of community service and health service organizations on why this deficit in implementation occurs are fundamental to understanding the solutions because these are the settings in which evidence-based interventions are implemented.<br>-Using concept mapping within a knowledge to action process can facilitate the engagement of multiple stakeholders in the utilization of LCS and in identifying next steps for action.
**47**	Vani N. Simmons et.al [43]	USA	to examine knowledge and attitudes about LDCT screening for lung cancer among an ethnically and racially diverse sample of high risk (HR) community members and primary care providers (PCP).	**Design**<br>Focus group discussion and content analysis.<br>**Participants**<br>Individuals and health care providers<br>**Sample size**<br>Individuals= N= 38<br>Providers= N=23<br>**Setting**<br>In person and telephone<br>**Gender**<br>Male and Female<br>**Age**<br>>=55	**Facilitators**<br>- knowledge.<br>- Recent change in insurance coverage.<br>**Barriers**<br>- Lack of knowledge<br>- Cost or insurance<br>- Fear of result<br>**Other risk factors**	**Conclusion:**<br>- Both High Risk participants and Primary Care Providers were not knowledgeable about LDCT.<br>- screening and recent changes in insurance coverage suggesting a potential educational opportunity.<br>**Implication:**<br>- Engaging both the medical community,<br>and those at increased risk for lung cancer is paramount for successful implementation.<br>-Understanding the barriers to lung screening across diverse community populations is necessary to

						improve screening rates and shared decision-making.<br>- Educational materials should be made available to Primary Care Providers and<br>patients for use in waiting rooms to increase awareness about LDCT.<br>-screening and to stimulate physician-patient communication and shared decision-making.<br>- Efforts are needed to educate Primary Care Providers about LCS guidelines and insurance coverage.<br>-Referral tools such as pop-up reminders and electronic forms with a list of pre-identified sites and radiologists certified in LDCT screening would facilitate the referral process.
**48**	Roberto Cardarelli et al, 2015[44]	USA	To Identifying Community Perspectives for a Lung Cancer Screening Awareness Campaign.	**<u>Design</u>**<br>Focus group discussion and content analysis.<br>**<u>Participants</u>**<br>Individuals<br>**<u>Sample size</u>**<br>N= 54<br>**<u>Setting</u>**<br>Face to face interview<br>**<u>Gender</u>**<br>Female=51%<br>Male=39%<br>**<u>Age</u>**<br>55-77	**<u>Facilitators</u>**<br><br>**<u>Barriers</u>**<br>- No information about LCS<br>- Lack of information access<br>**<u>Other risk factors</u>**<br>None	**<u>Conclusion</u>**<br>Most participants had not heard of lung<br>cancer screening. Cited needs for content of a campaign included benefits of early detection and payment information.<br>**<u>Implication</u>**<br>Messages considered most persuasive were those that include personal testimony, messages of hope, prolonged life, and an<br>emphasis on family and the ambition to survive. Having information come from one's family doctor or specialty provider was considered important to message communication.

						Messages about survivorship, family, and prolonged life should be considered in lung cancer screening awareness campaigns. Our results provide community input about messages regarding screening options.
**49**	Sonja Kumar et al,2020 [45]	UK	To explore the range of psychological and behavioral responses to LDCT screening offered as part of a Lung Heath Check (LHC).	**<u>Design</u>**<br>Semi-structured interview and framework analysis.<br>**<u>Participation</u>**<br>individuals<br>**<u>Sample size</u>**<br>N=23<br>**Setting**<br>Face to face and via telephone<br>**Gender**<br>Male=54%<br>Female=46%<br>**Age**<br>60-75	**<u>Facilitators</u>**<br>None<br>**<u>Barriers</u>**<br>✓ Existing concerns about lung health and smoking history<br>✓ Social support<br>✓ Stigma and self-blame<br>✓ Negativity and fatalism<br>✓ Competing priorities<br>**<u>Other risk factors</u>**	**<u>Conclusion</u>**<br>✓ Both psychologically and behaviorally, should direct a broader research agenda to ensure all stages of screening delivery and communication are designed to promote well-being, motivate positive behavior change and maximize patient benefits.<br>**<u>Implication</u>**<br>▪ Positive behavioral responses extended beyond smoking cessation to include anticipated implications for other cancer prevention and early detection behaviors, such as symptom presentation.
**50**	Rodríguez-Rabassa, et al,2020 [46]	USA	To investigated knowledge and attitudes about LDCT in focus groups of primary care physicians (PCP) and individuals at high-risk for lung Cancer.	**<u>Design</u>**<br>FGD and content analysis using constant comparison method analysis.<br>**<u>Participation</u>**<br>Individuals and providers<br>**<u>Sample size</u>**<br>N=37<br>**<u>Setting</u>**<br>Face to face<br>**<u>Gender</u>**<br>Male= 46%<br>Female=54%<br>**<u>Age</u>**<br>55 to 80 years old	**<u>Facilitators</u>**<br>- limited knowledge of participant about LDCT<br>- Concerns regarding insurance coverage.<br>-never had a provider recommend LDCT<br>--Participants believed that having symptoms was necessary to obtain LDCT screening.<br>- few Primary care physicians had ever recommended LDCT to a patient<br>**<u>Barriers</u>**	**<u>Conclusion</u>**<br>-Education on LDCT is needed and Preventive Services Task Force guidelines should be widely distributed to encourage physician recommendations.<br>**<u>Implication</u>**<br>-The development of targeted educational materials (promotion strategy and education message would help Primary Care Physicians and High-Risk Individuals increase awareness about LDCT

					- Fears about results and the procedure.<br>- Challenges with insurance.<br>-Lack of knowledge.<br>**Other factors**	screening and encourage communication between physicians and patients and facilitate the process of shared decision-making, thus reducing lung cancer mortality and to attract a diverse cohort of High-Risk Individuals to lung cancer screening.<br>Increased awareness of clinical guidelines, counseling, and shared decision-making visits are needed to increase utilization of LDCT for early detection.
**51**	Patel, D.et al 2012[47]	UK	To explore acceptability of the methods of screening and reasons for participation and non-participation in the Lung-SEARCH trial. (What influences the decision to take part in a lung cancer screening program?)	**Design**<br>Semi-structured interviews<br>**Participants**<br>Individuals<br>**Sample size**<br>N=60<br>**Setting**<br>At home (face to face) and via telephone<br>**Gender**<br>Male : Female<br>**Age**<br>52-81 (average 54)	**Facilitators**<br>- The value of life and perceptions of age<br>- Altruism<br>**Barriers**<br>-Fear of bronchoscopy<br>- inconvenience of travelling to hospitals for screening investigations and perceive themselves as having low susceptibility to lung cancer or being too old to benefit.<br>- Poor knowledge and mis understanding (too old to be bothered, 'worriers', 'fatalists', and 'avoiders).<br>**Other factors**	**Conclusion**<br>▪ Decision to participate or decline reflects a complex balance of factors including acceptability and convenience of screening methods, risk perception, altruism, and self-interest.<br>**Implication**<br>▪ Improving practical and changing cognitive aspects of participation will be key to improving uptake of LCS.<br>▪ For research, further work could explore. how people perceive risk and how this is related to ageing, and the role of fatalism in the health behavior of smokers.<br>▪ For screening programs and trials, if bronchoscopy is found to be useful as part of a screening program, more work needs to be done to present it as an acceptable screening tool.

						▪ Attempts to maximize participation in LCS programs should recognize that decisions to take part involve complex judgements by patients. These judgements reflect practical issues (such as getting to hospital, acceptability of bronchoscopy) and cognitive (risk/benefit) judgements, the latter often affected by personal traits and experience.
**52**	Herb, J.et al, 2023. [3]	USA	to explore the patient-perceived barriers and facilitators to care for suspected or newly diagnosed early-stage	<u>**Design**</u><br>✓ Semi-structured interviews and content analysis.<br><u>***Participants***</u><br>✓ *Adult patients*<br><u>***Sample size***</u><br>✓ N=26<br><u>**Setting**</u><br>✓ tertiary public academic referral center and home to the Lineberger Comprehensive Cancer Center<br><u>**Gender**</u><br>▪ Male (38%)<br>▪ Female (62%)<br><u>**Age**</u><br>▪ mean age= 62 yrs. (SD: 8.4 years)	<u>**Facilitator**</u><br><u>**Barriers**</u><br>▪ Trust with providers and health systems and patient self-advocacy, financial toxicity, treatment experience; social constraints magnified, provider advocacy, care coordination and good communication.<br><u>**Other factors**</u><br>-----	<u>**Conclusion**</u><br>▪ Needs to address these factors to improve quality of care among lung cancer patients.<br><u>**Implication**</u><br>1). Primary care providers can play a critical role in the treatment process.<br>✓ By encouraging patients to receive LCS<br>✓ By advocating for and increasing patient's comfort with the health system throughout the time from diagnosis to treatment.<br>▪ Patients who trust their primary care providers can benefit greatly from proactive providers who provide direct referrals to surgeons and oncologists.<br>2). Patients who are vulnerable due to socioeconomic factors could benefit from more direct interventions to identify existing financial need and active navigation programs to help circumvent other

			common barriers such as transportation and obtaining financial assistance.

Table S4. Themes for facilitators to lung cancer screening.

**Patient Individual-Level**	**Interpersonal- Level (Provider-Patient)**	**Cultural Level Barriers**	**Organizational Level (Institution/Policy)**
-education<br>-<br>-	- Receiving a screening recommendation from a healthcare provider<br>-<br>-	- The value of life and perceptions of age<br>-<br>-	- A decision-making aid<br>-<br>-

Table S5. Themes for barriers to lung cancer screening.

**Barriers**				
Individual level		Interpersonal- Level (Provider-Patient)	Cultural Level Barriers	Organizational-Level (institution/policy)
Patient Level	Provider Level			
❖ **Knowledge/Capacity**<br>-<br>-<br>-<br>❖ **Behavior/Attitude**	❖ Education<br>-<br>-<br>❖ Pitfalls<br>-<br>-<br>❖ Processes	❖ Patient-Provider Relationship<br>-<br>-<br>❖ Communication Quality<br>-<br>-	-<br>-<br>-	❖ Access to Resources<br>-<br>-<br>-<br>❖ Care Coordination:<br>-<br>-

-<br>-<br>-<br>❖ **Comorbidities**<br>-<br>-<br>-<br>❖ **Perception**<br>-<br>-<br>-	-<br>-<br>❖ **Policy**<br>-<br>-			-<br>❖ Implementing LCS:<br>-<br>-<br>-<br>❖ Experiences of accessing cancer screening and diagnosis<br>-<br>-<br>-<br>❖ Challenge to implement LCS<br>-<br>-<br>-

Reference

1. Abubaker-Sharif, M., et al., *Primary care physician perceptions of shared decision making in lung cancer screening.* Journal of Cancer Education, 2020: p. 1-9.
2. Brown, L.R., et al., *Increasing uptake to a lung cancer screening programme: building with communities through co-design.* BMC Public Health, 2022. **22**(1): p. 1-17.
3. Herb, J., et al., *Barriers and facilitators to early-stage lung cancer care in the USA: a qualitative study.* Supportive Care in Cancer, 2023. **31**(1): p. 21.
4. Carter-Harris, L., et al., *A qualitative study exploring why individuals opt out of lung cancer screening.* Family practice, 2017. **34**(2): p. 239-244.
5. Crothers, K., et al., *Patients' attitudes regarding lung cancer screening and decision aids. A survey and focus group study.* Annals of the American Thoracic Society, 2016. **13**(11): p. 1992-2001.
6. Gressard, L., et al., *A qualitative analysis of smokers' perceptions about lung cancer screening.* BMC public health, 2017. **17**(1): p. 1-8.
7. Hoffman, R.M., et al., *Peer Reviewed: Attitudes and Beliefs of Primary Care Providers in New Mexico About Lung Cancer Screening Using Low-Dose Computed Tomography.* Preventing chronic disease, 2015. **12**.
8. Melzer, A.C., et al., *What exactly is shared decision-making? A qualitative study of shared decision-making in lung cancer screening.* Journal of general internal medicine, 2020. **35**(2): p. 546-553.
9. Mejia, M.C., et al., *Key informants' perspectives on implementing a comprehensive lung cancer screening program in a safety net healthcare system: Leadership, successes, and barriers.* Journal of Cancer Education, 2022. **37**(4): p. 1144-1151.
10. Mishra, S.I., et al., *Peer reviewed: Patient perspectives on low-dose computed tomography for lung cancer screening, New Mexico, 2014.* Preventing chronic disease, 2016. **13**.
11. Sin, M.-K., A. Ha, and V. Taylor, *Sociocultural barriers to lung cancer screening among Korean immigrant men.* Journal of community health, 2016. **41**(4): p. 790-797.

12. Schiffelbein, J.E., et al., *Barriers, facilitators, and suggested interventions for lung cancer screening among a rural screening-eligible population.* Journal of Primary Care & Community Health, 2020. **11**: p. 2150132720930544.
13. Sharf, B.F., L.A. Stelljes, and H.S. Gordon, *'A little bitty spot and I'm a big man': patients' perspectives on refusing diagnosis or treatment for lung cancer.* Psycho-oncology, 2005. **14**(8): p. 636-646.
14. Wiener, R.S., et al., *Patient and clinician perspectives on shared decision-making in early adopting lung cancer screening programs: a qualitative study.* Journal of general internal medicine, 2018. **33**(7): p. 1035-1042.
15. Williams, R.M., et al., *Lung cancer screening decisional needs among African American smokers of lower socioeconomic status.* Ethnicity & health, 2022. **27**(3): p. 565-583.
16. Ali, N., et al., *Barriers to uptake among high-risk individuals declining participation in lung cancer screening: a mixed methods analysis of the UK Lung Cancer Screening (UKLS) trial.* BMJ open, 2015. **5**(7): p. e008254.
17. Carter-Harris, L., et al., *Lung cancer screening: what do long-term smokers know and believe?* Health Expectations, 2017. **20**(1): p. 59-68.
18. Greene, P.A., et al., *Challenges to educating smokers about lung cancer screening: A qualitative study of decision making experiences in primary care.* Journal of Cancer Education, 2019. **34**(6): p. 1142-1149.
19. Draucker, C.B., et al., *Understanding the decision to screen for lung cancer or not: A qualitative analysis.* Health Expectations, 2019. **22**(6): p. 1314-1321.
20. Lowenstein, M., et al., *Real-world lung cancer screening decision-making: Barriers and facilitators.* Lung Cancer, 2019. **133**: p. 32-37.
21. Quaife, S.L., et al., *Attitudes towards lung cancer screening in socioeconomically deprived and heavy smoking communities: informing screening communication.* Health Expectations, 2017. **20**(4): p. 563-573.
22. Roth, J.A., et al., *A qualitative study exploring patient motivations for screening for lung cancer.* PloS one, 2018. **13**(7): p. e0196758.
23. Dhavale, P., A. Koparkar, and P. Fernandes, *Palliative care interventions from a social work perspective and the challenges faced by patients and caregivers during COVID-19.* Indian Journal of Palliative Care, 2020. **26**(Suppl 1): p. S58.
24. Hyland, K.A. and H.S. Jim, *Behavioral and psychosocial responses of people receiving treatment for advanced lung cancer during the COVID-19 pandemic: A qualitative analysis.* Psycho-oncology, 2020.
25. Guven, D.C., et al., *Perspectives, knowledge, and fears of cancer patients about COVID-19.* Frontiers in oncology, 2020. **10**: p. 1553.
26. Schellekens, M.P. and M.L. van der Lee, *Loneliness and belonging: exploring experiences with the COVID-19 pandemic in psycho-oncology.* Psychooncology, 2020. **29**(9): p. 1399-1401.
27. Mazzone, P.J., et al., *Management of lung nodules and lung cancer screening during the COVID-19 pandemic: CHEST expert panel report.* Chest, 2020. **158**(1): p. 406-415.
28. Croswell, J.M., et al., *Cancer screening in the US through the COVID-19 pandemic, recovery, and beyond.* Preventive Medicine, 2021. **151**: p. 106595.
29. Quaife, S.L., et al., *Smokers' interest in a lung cancer screening programme: a national survey in England.* BMC cancer, 2018. **18**(1): p. 1-10.
30. Klarenbeek, S.E., et al., *Barriers and facilitators for implementation of a computerized clinical decision support system in lung cancer multidisciplinary team meetings—a qualitative assessment.* Biology, 2020. **10**(1): p. 9.

31. Kanodra, N.M., et al., *Primary care provider and patient perspectives on lung cancer screening. A qualitative study.* Annals of the American Thoracic Society, 2016. **13**(11): p. 1977-1982.
32. Holman, A., et al., *Patient perspectives on longitudinal adherence to lung cancer screening.* Chest, 2022.
33. Richman, I.B., T.V. Prasad, and C.P. Gross, *Lost to follow up?: A qualitative study of why some patients do not pursue lung cancer screening.* Preventive Medicine Reports, 2022. **29**: p. 101909.
34. Dunlop, K.L., et al., *Motivation is not enough: A qualitative study of lung cancer screening uptake in Australia to inform future implementation.* Plos one, 2022. **17**(9): p. e0275361.
35. Tod, A.M., J. Craven, and P. Allmark, *Diagnostic delay in lung cancer: a qualitative study.* Journal of advanced nursing, 2008. **61**(3): p. 336-343.
36. Hall, S.a.E., et al., *Lung cancer: an exploration of patient and general practitioner perspectives on the realities of care in rural Western Australia.* Australian Journal of Rural Health, 2008. **16**(6): p. 355-362.
37. Walton, L., et al., *Patient perceptions of barriers to the early diagnosis of lung cancer and advice for health service improvement.* Family practice, 2013. **30**(4): p. 436-444.
38. Sayani, A., et al., *Perspectives of family physicians towards access to lung cancer screening for individuals living with low income–a qualitative study.* BMC family practice, 2021. **22**(1): p. 1-9.
39. Sayani, A., et al., *Advancing health equity in cancer care: the lived experiences of poverty and access to lung cancer screening.* PloS one, 2021. **16**(5): p. e0251264.
40. Hanna, K., et al., *Cancer Screening Among Rural and Urban Clinics During COVID-19: A Multistate Qualitative Study.* JCO Oncology Practice, 2022: p. OP. 21.00658.
41. Margariti, C., et al., *Healthcare professionals' perspectives on lung cancer screening in the UK: a qualitative study.* BJGP open, 2020. **4**(3).
42. Lobb, R., A.D. Pinto, and A. Lofters, *Using concept mapping in the knowledge-to-action process to compare stakeholder opinions on barriers to use of cancer screening among South Asians.* Implementation Science, 2013. **8**(1): p. 1-12.
43. Simmons, V.N., et al., *High-risk community and primary care providers knowledge about and barriers to low-dose computed topography lung cancer screening.* Lung Cancer, 2017. **106**: p. 42-49.
44. Cardarelli, R., et al., *Identifying community perspectives for a lung cancer screening awareness campaign in Appalachia Kentucky: the Terminate Lung Cancer (TLC) study.* Journal of Cancer Education, 2017. **32**(1): p. 125-134.
45. Kummer, S., et al., *Mapping the spectrum of psychological and behavioural responses to low-dose CT lung cancer screening offered within a Lung Health Check.* Health Expectations, 2020. **23**(2): p. 433-441.
46. Rodríguez-Rabassa, M.S., et al., *Perceptions of and barriers to lung cancer screening among physicians in Puerto Rico: a qualitative study.* Journal of health care for the poor and underserved, 2020. **31**(2): p. 973.
47. Patel, D., et al., *Attitudes to participation in a lung cancer screening trial: a qualitative study.* Thorax, 2012. **67**(5): p. 418-425.
