# Supplementary figures and images for: Facilitators and Barriers to Lung Cancer Screening during Long COVID: A Global Systematic Review and Meta-Study Synthesis of Qualitative Research
Source: Int J Environ Res Public Health. 2024 Apr 25;21(5):534. doi: 10.3390/ijerph21050534 (PMC11121223; doi:10.3390/ijerph21050534)

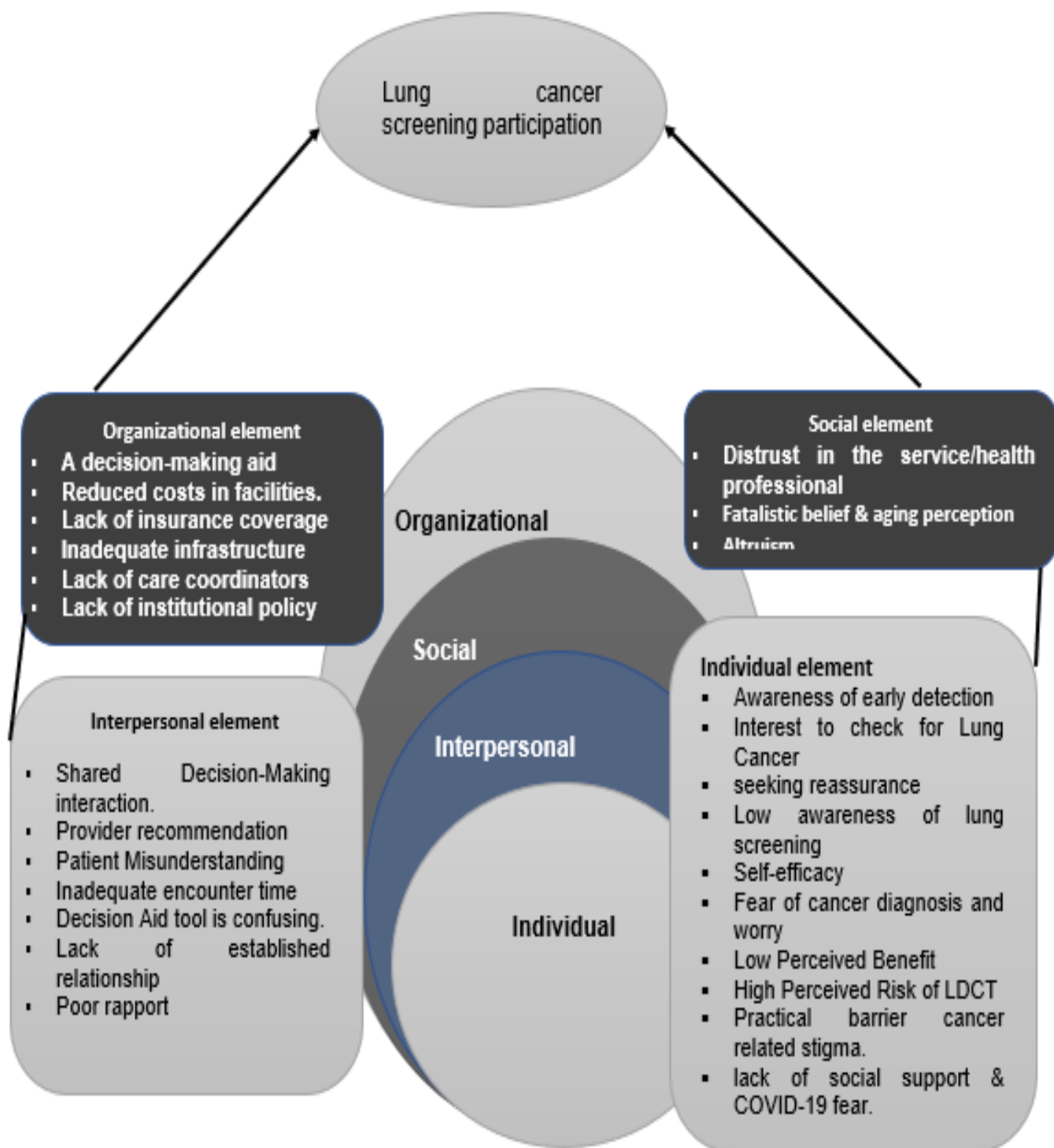

**Figure S1.** A conceptual framework of factor influence to participate lung cancer screening.

Supplement: Supplementary file 1 [file ijerph-21-00534-s001.zip › Figure S1.pdf]
